# Survival predictors after intubation in medical wards: A prospective study in 151 patients

**DOI:** 10.1371/journal.pone.0234181

**Published:** 2020-06-01

**Authors:** Dimitrios Basoulis, Stavros Liatis, Marina Skouloudi, Konstantinos Makrilakis, Georgios L. Daikos, Petros P. Sfikakis

**Affiliations:** 1 First Department of Propaedeutic Internal Medicine, Laiko General Hospital, National and Kapodistrian University of Athens, Athens, Greece; 2 First Department of Internal Medicine, Laiko General Hospital, National and Kapodistrian University of Athens, Athens, Greece; European University Cyprus, CYPRUS

## Abstract

**Introduction:**

In health care systems in need of additional intensive care unit (ICU) beds, the decision to mechanically ventilate critically ill patients in Internal Medicine (IM) Department wards needs to balance patients’ health outcomes, possible futility, and logistics. We aimed to examine the survival rates and predictors in these patients.

**Methods:**

We prospectively enrolled consecutive patients receiving mechanical ventilation during their care in the IM wards of a tertiary University hospital between April 2016 and December 2018. Primary outcome was 90-day mortality and secondary outcomes were in-hospital mortality and ICU transfer.

**Results:**

Our cohort consisted of 151 unique patient intubations, of whom 74 (49%) patients were transferred to ICU within a median of 0 days (range 0–7). Compared to patients who remained in the wards, patients transferred to ICU had lower in-hospital and 90-day mortality (65% vs. 97%, and 70% vs. 99%, respectively, p<0.001 for both). Amongst several possible predictors of survival in the ICU, sequential organ failure assessment (SOFA) score at the time of intubation had the best prognostic accuracy with an AUROC of 0.818 and 0.855 for in-hospital and 90-day mortality, respectively. A baseline SOFA score ≤8 had a 100% sensitivity for survival prediction in ICU. However, out of 26 patients with SOFA score ≤8 who remained in the wards, only one survived, whereas 19 patients with SOFA score >8 who were transferred to ICUs received futile care.

**Conclusion:**

Mortality for patients receiving mechanical ventilation in IM wards is almost inevitable when ICU availability is lacking. Therefore, applying additional transfer criteria beyond the SOFA score is imperative.

## Introduction

Every physician caring for acutely ill patients may have to make decisions and recommendations regarding treatments that are considered as potentially futile at some point in their medical careers. In an era of technological bounds and leaps, our ability to sustain life has drastically improved, but in certain cases this life is dependent on artificial measures or the outcome is one of poor quality [1]. More than others, the decision to mechanically ventilate is crucial in determining survival if there is a chance for that, but in the cases of futile treatment it only serves to extend the agony and the grieving period of the patient’s family [2].

Aside from ethical dilemmas regarding the patient and his/her family, one needs to consider the principle of utilitarianism, i.e. medical decisions need to benefit the greatest possible number of people. The necessity to limit healthcare expenditure in order to provide the best available treatments to the majority of the people in need, means that futile treatments should be limited [3]. Several studies in Europe and the US have demonstrated that Intensive Care Units (ICU) are both understaffed and lacking in beds to cover the ever-increasing needs [4–7]. This is especially true for Greece that has been plagued by financial difficulties during the past years [8].

Within the Greek National Health Care System, the vast majority of hospitalized patients who are at some point in need of mechanical ventilation (MV) are intubated in the wards before their transfer to an ICU. The availability of ICU beds though is not adequate to cover the needs of all intubated patients at every point in time. The medical team that decides to proceed with MV is responsible for reporting this event to the National Center for Emergency Care (NCEC) as well as to the hospital’s ICU, where the patient is transferred if an available bed exists. Otherwise, the NCEC notifies daily all public- and private-sector ICUs about the list of intubated patients in need of an ICU bed, and a decision is made on a daily basis by the ICU physicians, based on bed availability and patient characteristics (long-term life expectancy, etc), as well as capabilities of the Unit, about the transfer of a patient to the ICU.

Lack of concise legislation regarding end of life treatment as well as prevalent religious views lead often to deciding to mechanically ventilate patients for whom such invasive measures might be deemed inappropriate. A study on the issue of withholding or withdrawing life support measures in Greek ICUs, showed that Greek intensivists are withholding cardiopulmonary resuscitation (CPR) but rarely other modalities. [9] In medical wards, the experience of most physicians in matters of palliative and critical care is limited. Palliative medicine is not a discrete specialty in Greece and internists are required a 3-month training period in the ICU to complete their medical specialty training in Internal Medicine (IM).

The goal of the current study was to record the health outcomes of patients who receive MV in medical wards of a tertiary care hospital in Athens, Greece, in order to reach conclusions regarding predictors of mortality for these patients. Given that some patients would eventually be transferred to an ICU, per the described process, a secondary goal was to describe the patient characteristics that would make this transfer more likely, since it is expected that the patients who get transferred have a better prognosis than those treated, for the duration of hospitalization, in medical wards.

## Materials and methods

### Study design and outcomes

This is an analysis of prospectively collected observational data from consecutive patients placed on MV during hospitalization in the two participating Internal Medicine departments of the Hospital. The primary outcome was 90-day post-intubation mortality. Secondary outcomes included in-hospital mortality and transfer to an ICU.

The study was approved by the Laiko General Hospital Scientific and Ethics Review Board (protocol number: 212/04-03-2016); informed consent was waived as collected data was anonymized and there was no intervention or treatment performed on the patients included.

### Study population

The total recruiting period was 33 months (from 1^st^ April 2016 to 31^st^ December 2018) and 10 months (from 1^st^ June 2017 to 1^st^ April 2018) for the two participating departments respectively. All patients fulfilling all inclusion criteria and none of the exclusion criteria were included in the study.

The inclusion criteria were: a) age >18 years, b) placement under mechanical ventilation while in the care of physicians working in the participating IM wards.

The exclusion criteria were: a) placement under mechanical ventilation while in the care of physicians not working in the participating IM wards with subsequent transfer of the patient to these wards.

### Measurements

We recorded demographic data (sex, age, marital status, socioeconomic status), medical history (information about Charlson score [10] and frailty, based on the Canadian Study of Health and Aging (CSHA) frailty scale [11]), clinical and laboratory data on the day of intubation (in order to calculate the following scores: Acute Physiology Assessment and Chronic Health Evaluation (APACHE) II, III and IV, Simplified Acute Physiology Score (SAPS) II and III, Sequential Organ Failure Assessment (SOFA), and Mortality Prediction Model (MPM) II and III for day 0) [12–19].

### Statistical analysis

Descriptive statistics are presented as counts (%) for categorical variables and as medians (25^th^–75^th^ percentile) for non-normally distributed continuous variables or as means ± standard deviation (SD) for normally distributed continuous variables. Normality of distribution was examined using the Kolmogorov–Smirnov test. Univariate and multivariate analyses were performed using the Cox proportional hazards regression model for both 90-day and in-hospital mortality. For the ICU transfer secondary outcome, a binary logistic regression model was employed. Variables in the multivariate model, were entered hierarchically, and all variables with statistical significance, as defined by a p<0.05 in the univariate analysis, were included. Results of the Cox model are presented as Hazard Ratios (HR), while results of the logistic regression as Odds Ratios (OR), both with 95% confidence intervals (CI) and with a statistical significance for p<0.05. The analysis was performed using SPSS Statistics for Windows, Version 25.0 (2017, Armonk, NY, IBM Corp.).

## Results

### Baseline characteristics

A total of 157 intubations were recorded in 151 patients (6 patients were intubated for a second time during the same hospitalization; subsequently, only the first events were included in analyses), with an incidence of 3.11 intubations/1000 patient-days and a rate of 3.74 intubations/month.

The median age of the study population was 72 years (58–80) and 51% were male. The median time from admission to MV was 2 days (0–7) and the median duration of MV was 4 days (1–10). Half of the patients (74/151) were transferred to an ICU, 51.4% (38/74) on the day they were intubated, and 33.8% (25/74) the next day. No patient was admitted to the ICU after one week from their intubation had passed. A quarter of our population was placed under MV at the Emergency Department (25.2%, 38/151), 68.9% (104/151) at the Internal Medicine wards, while 6% (9/151) were intubated in other locations, such as the dialysis or endoscopy units. Sixty-five percent were intubated during weekdays (98/151). Finally, 23.8% (36/151) were intubated during the morning shift, 41.7% (63/151) during normal night shifts, and 34.4% (52/151) during “on-call” night shifts. Baseline demographic and clinical parameters are shown in Table 1.

**Table 1:** Baseline demographic and clinical characteristics of the patient population.

Variable	n/N(%) or mean±SD or median (IQR 25–75)
**Patient Characteristics**	
**Female gender**	74/151 (49%)
**Age, years**	72/151 (58–80)
With spouse**	83/142 (55%)
With offspring**	109/148 (72.2)
**Charlson score**	6 (4–7)
**Intubation Information**	
**Main Indication**	
**Respiratory**	63/151 (41.7)
**Neurological**	54/151 (35.8)
**Cardiac arrest**	34/151 (22.5)
**Location**	
**Emergency Dpt**	38/151 (25.2)
**Ward**	104/151 (68.9)
**Other**	9/151 (6)
**On weekdays**	98 (64.9)
**Hospital status**	
**Normal night**	52/151 (34.4)
**On-call night**	63/151 (41.7)
**Morning shift**	36/151 (23.8)
**Emergency indication**	100/151 (66.2)
**Circulatory support**	35/151 (23.2)
**Infection**	
**No infection**	91/151 (60.3)
**Community**	30/151 (19.9)
**Nosocomial**	30/151 (19.9)
**Septic Shock**	21/151 (13.9)
**Vital signs and laboratory values immediately before intubation**	
**Heart rate (/min)**	100 (85–115)
**MAP (mmHg)**	85 (61.7–96.7)
**Temperature (°C)**	36.6 (36.2–37.7)
**RR (/min)**	28 (16.8–35)
**Blood pH**	7.3 (7.16–7.4)
**PO_2_/FiO_2_**	125 (81.25–257.25)
**GCS**	8 (3–14)
**Henatocrit (%)**	32.7 (26.8–38.3)
**White blood cells (x 10^9^/L)**	11.11 (5.74–18.1)
**Neutropenia**	5/151 (1)
**Platelet count (x 10^9^/L)**	163.5 (75–250.3)
**>150**	86/151 (57)
**100–149**	20/151 (13.2)
**50–99**	15/151 (9.9)
**20–49**	17/151 (11.3)
**<20**	13/151 (8.6)
**Serum Creatinine (μmnol/L)**	106.1 (70.7–203.3)
**Serum Sodium (mmol/L)**	140 (136–145)
**Serum Potassium (mmol/L)**	4.3 (3.8–4.9)
**Serum Bilirubin (μmol/L)**	11.1 (7–20.9)
**Serum Glucose (mmol/L)**	7.94 (5.77–10.66)
**Serum Albumin (g/L)**	30.8 ± 7.5
**Predictive Scores**	
**APACHE II**	26 (21–31)
**APACHE III**	94 (77–124)
**APACHE IV**	86 (69–111)
**SAPS II**	58 (44–70)
**SAPS III**	79 (67–90)
**SOFA**	8 (5–11)
**MPM II Day 0 mortality (%)**	56.6 (30.5–80.1)
**MPM III Day 0 mortality (%)**	61.4 (28.3–86.7)
**Transfer to ICU**	74/151 (49)

**Some data is missing because some patients did not stay in the hospital long enough to collect information regarding their family status. Categorical variables presented as n/N (%), continuous variables presented as mean ± SD or median [25th–75th percentile]. Ref: reference, GCS: Glasgow Coma Scale, Dpt: Department, ICU: Intensive Care Unit, MAP: Mean arterial pressure, RR: respiratory rate, APACHE: Acute Physiology Assessment and Chronic Health Evaluation, SAPS: Simplified Acute Physiology Score, SOFA: Sequential Organ Failure Assessment, MPM: Mortality Prediction Model.

### Indications for mechanical ventilation

Based on the indication for intubation, 41.7% (63/151) were intubated due to respiratory failure, 35.8% (54/151) due to decreased level of consciousness to preserve their airway and 22.5% (34/151) following a cardiac arrest. Based on International Classification of Diseases 10 (ICD-10) diagnoses, reported by the attending physicians on the day of intubation, 52.9% (80/151) were placed on mechanical ventilation for infectious causes, 10.6% (16/151) due to heart conditions, 21.2% (31/151) due to neurological conditions, 8.6% (13/151) due to respiratory problems and 7.3% (11/151) due to other causes.

### Predictors of 90-day survival

**Fig 1:**
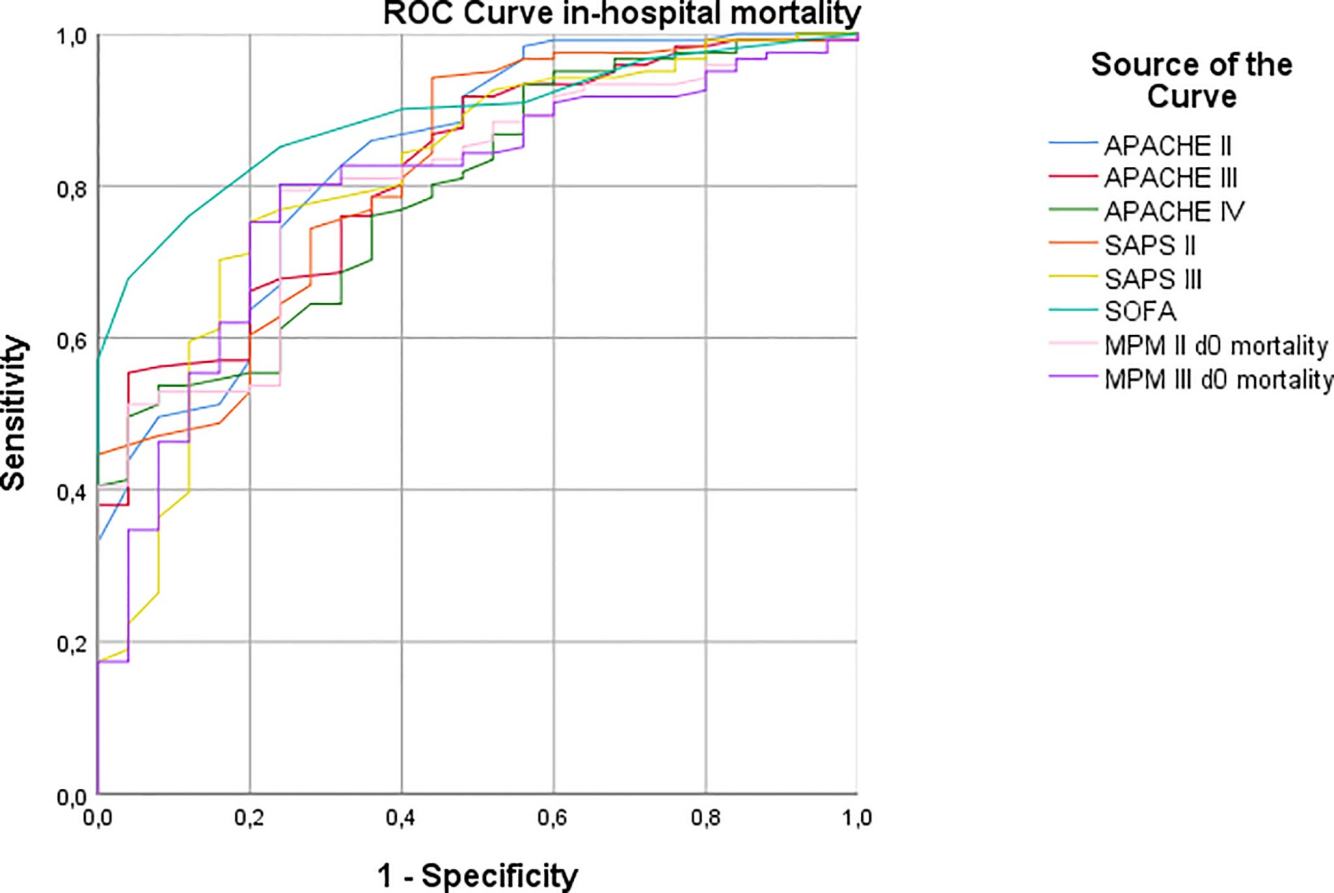
ROC curve analysis. In-hospital mortality ROC curve of the tested predictive scores. APACHE: Acute Physiology Assessment and Chronic Health Evaluation, SAPS: Simplified Acute Physiology Score, SOFA: Sequential Organ Failure Assessment, MPM: Mortality Prediction Model.

**Fig 2:**
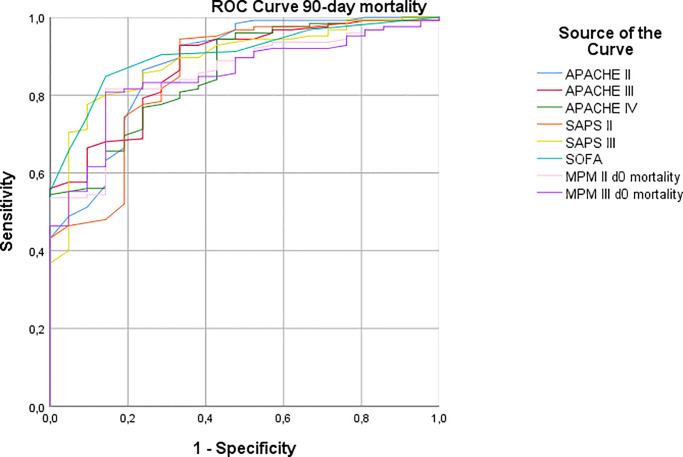
ROC curve analysis. 90-day mortality ROC curve of the tested predictive scores. APACHE: Acute Physiology Assessment and Chronic Health Evaluation, SAPS: Simplified Acute Physiology Score, SOFA: Sequential Organ Failure Assessment, MPM: Mortality Prediction Model.

Mortality at 90 days was 84.7% (127/150, one patient was lost to follow-up after discharge). Mortality analyses for the entire population are available in the supplementary material (S1–S4 Tables). Out of the predictive scores tested in the univariate analysis, SOFA presented the best diagnostic accuracy with an AUROC (area under the receiver operator characteristic curve) (Table 2, Figs 1 and 2). For this reason, in multivariate analysis (S3 and S4 Tables), we opted to use the SOFA score, as using them all at the same time would be redundant. In the hierarchical multivariate model for 90-day mortality, in the block where all the parameters at the time of the decision to intubate were included, higher Charlson score (aHR 1.16, 95%CI 1.07–1.27, p = 0.001), lower platelet count (aHR 0.997, 95%CI 0.995–0.999, p = 0.003) and higher SOFA score (aHR 1.16, 95%CI 1.05–1.28, p = 0.004) were significant predictors. When ICU transfer was included in the model, the only variables that retained statistical significance were higher SOFA score (aHR 1.16, 95%CI 1.05–1.29, p = 0.003) and ICU transfer (aHR 0.25, 95%CI 0.15–0.43, p<0.001).

**Table 2:** ROC curve comparison between the different prognostic scores.

Test	Area	p	95% CI	Cut-off	Sensitivity (%)	Specificity (%)
**In-Hospital mortality**						
**APACHE II**	0.836	<0.001	0.75–0.921	22	82.6	68
**APACHE III**	0.818	<0.001	0.736–0.899	81	78.5	64
**APACHE IV**	0.792	<0.001	0.705–0.878	74	76	64
**SAPS II**	0.818	<0.001	0.73–0.905	48	78.5	64
**SAPS III**	0.809	<0.001	0.712–0.905	71	75.2	80
**SOFA**	0.890	<0.001	0.835–0.943	6	85.1	76
**MPM II Day 0 mortality**	0.803	<0.001	0.719–0.886	35%	79.3	76
**MPM III Day 0 mortality**	0.794	<0.001	0.701–0.885	35%	80.2	76
**90-day mortality**						
**APACHE II**	0.879	<0.001	0.798–0.959	21	86.4	76.2
**APACHE III**	0.876	<0.001	0.805–0.945	81	79.2	76.2
**APACHE IV**	0.856	<0.001	0.779–0.933	74	76.8	76.2
**SAPS II**	0.854	<0.001	0.764–0.943	50	74.4	81
**SAPS III**	0.888	<0.001	0.82–0.955	70	77.6	90.5
**SOFA**	0.905	<0.001	0.85–0.959	6	84.8	85.7
**MPM II Day 0 mortality**	0.854	<0.001	0.78–0.926	34%	81.6	85.7
**MPM III Day 0 mortality**	0.848	<0.001	0.774–0.921	34%	80.8	85.7

**Fig 3:**
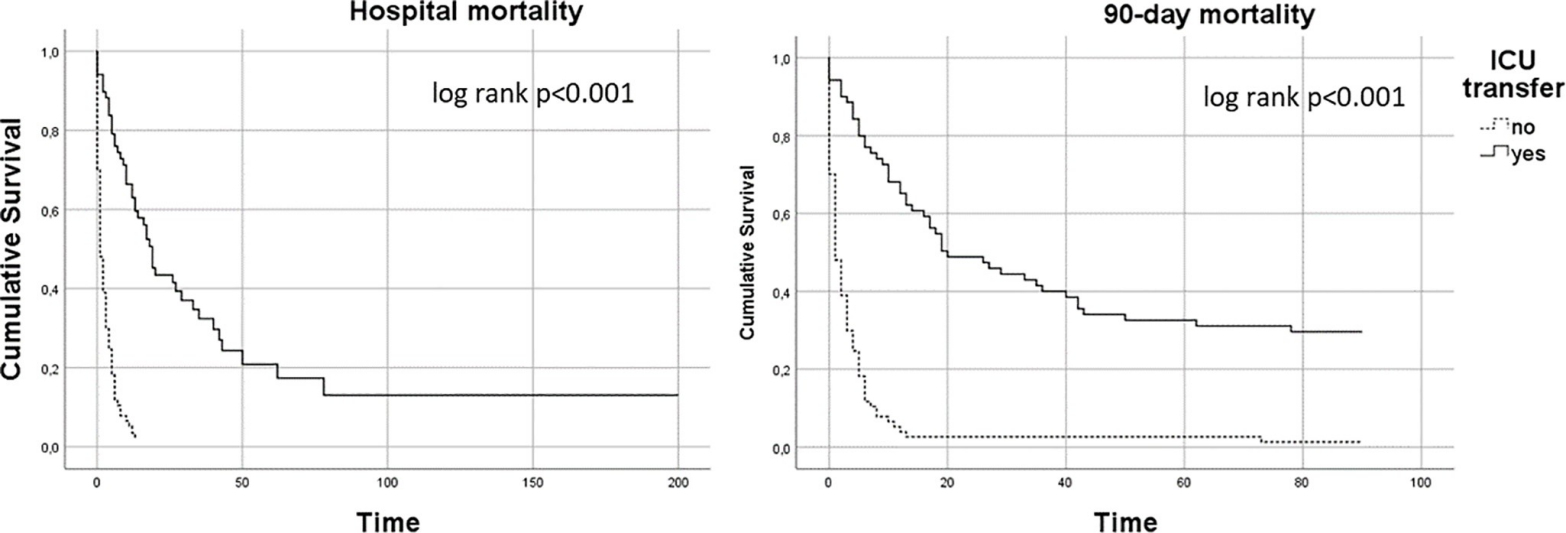
Kaplan Meier survival analysis. The dashed line represents the survival of the patients that were not transferred to the ICU and the continuous line those that were transferred.

Cut-off values were derived based on the ROC graphs (Figs 2 and 3). CI: Confidence interval, APACHE: Acute Physiology Assessment and Chronic Health Evaluation, SAPS: Simplified Acute Physiology Score, SOFA: Sequential Organ Failure Assessment, MPM: Mortality Prediction Model.

### Predictors of ICU transfer

A total of seventy-four (74/151, 49%) patients were transferred to an ICU. The parameters during which the intubation would predict an ICU transfer were investigated. Univariate analysis is presented in Table 3. Only those parameters that displayed statistical significance in the univariate analysis were included in the multivariate analysis (Table 4 and S5 Table). In this model, the only predictors for ICU transfer, were a higher GCS score (aOR 1.17, 95%CI 1.03–1.34, p = 0.02), the absence of cardiac arrest (aOR 0.13, 95%CI 0.03–0.46, p = 0.002) or a neurological indication for intubation (aOR 0.26, 95%CI 0.09–0.82, p = 0.021) and lower Charlson score (aOR 0.75, 95%CI 0.6–0.94, p = 0.012).

**Table 3:** Demographic, clinical, and laboratory characteristics of patients who were transferred and not transferred to ICU at the time of their intubation.

	Not transferred	Transferred	OR	95%CI	p
	N = 77	N = 74			
**Demographics**					
**Female gender**	39/77 (50.6)	35/74 (47.3)	0.87	0.46–1.66	0.68
**Age, years**	74 (61–82.5)	70 (56–78.25)	0.98	0.96–1	0.062
With spouse*	39/74 (52.7)	44/68 (64.7)	1.65	0.84–3.23	0.148
With offspring*	54/75 (72)	55/73 (75.3)	1.19	0.57–2.47	0.645
**Charlson score**	6 (5–8)	5 (2–7)	0.82	0.72–0.93	**0.002**
**Intubation information**					
**Main indication**					
**Respiratory**	19/77 (24.7)	44/74 (59.5)	ref	ref	ref
**Neurological**	33/77 (42.9)	21/74 (28.4)	0.28	0.13–0.59	**0.001**
**Cardiac arrest**	25/77 (32.5)	9/74 (12.2)	0.16	0.06–0.4	**<0.001**
**Location**					
**Emergency Dpt**	19/77 (24.7)	20/74 (27)	ref	ref	ref
**Ward**	55/77 (71.4)	49/74 (66.2)	0.82	0.39–1.72	0.594
**Other**	4/77 (5.2)	5/74 (6.8)	1.13	0.26–4.85	0.874
**On Weekdays**	46/77 (59.7)	52/74 (70.3)	1.59	0.81–3.13	0.177
**Hospital status**					
**Normal night**	27/77 (35.1)	25/74 (33.8)	ref	ref	ref
**On-call night**	32/77 (41.6)	31/74 (41.9)	1.05	0.5–2.18	0.904
**Morning shift**	18/77 (23.4)	18/74 (24.3)	1.08	0.46–2.53	0.859
**Emergency indication**	55/77 (71.4)	45/74 (60.8)	0.62	0.31–1.23	0.169
**Circulatory support**	22/77 (28.6)	13/74 (17.6)	0.53	0.25–1.16	0.112
**Infection**					
**No infection**	41/77 (53.2)	50/74 (67.6)	ref	ref	ref
**Community**	16/77 (20.8)	14/74 (18.9)	0.72	0.31–1.64	0.432
**Nosocomial**	20/77 (26)	10/74 (13.5)	0.41	0.17–0.97	**0.043**
**Septic Shock**	11/77 (14.3)	10/74 (13.5)	0.94	0.37–2.36	0.891
**Vital signs and laboratory values immediately before intubation**					
**Heart rate (bpm)**	100 (85–116)	100 (85.5–115)	1.01	0.99–1.02	0.485
**MAP (mmHg)**	80 (56.7–97.2)	86.7 (67.5–96.7)	1.01	1–1.02	0.114
**Temperature (°C)**	36.6 (36–38.1)	36.6 (36.2–37.2)	0.79	0.61–1.04	0.089
**RR (/min)**	28 (15–35)	30 (17–35)	1	0.97–1.02	0.906
**Blood pH**	7.32 (7.19–7.41)	7.27 (7.15–7.4)	0.54	0.08–3.48	0.515
**PO_2_/FiO_2_**	136 (83–268)	123.3 (75–247.5)	0.998	0.996–1.002	0.568
**GCS**	6 (3–12.5)	10 (4.75–15)	1.09	1.02–1.17	**0.015**
**Henatocrit (%)**	30.2 (25.2–37.9)	34.1 (28.8–38.9)	1	0.99–1.01	0.767
**White blood cells (x 10^9^/L)**	9.37 (4.23–17.47)	12.65 (7.29–18.27)	1	0.99–1.01	0.961
**Neutropenia**	4/77 (5.2)	1/74 (1.4)	0.25	0.03–2.26	0.215
**Platelets (x 10^9^/L)**	111 (43.75–201)	213 (138–75–285.5)	1.007	1.004–1.01	**<0.001**
**>150**	32/77 (41.6)	54/74 (73)	ref	ref	ref
**100–149**	12/77 (15.6)	8/74 (10.6)	0.38	0.14–1.04	0.059
**50–99**	10/77 (13)	5/74 (6.8)	0.29	0.09–0.92	**0.035**
**20–49**	13/77 (16.9)	4/74 (5.4)	0.18	0.05–059	**0.005**
**<20**	10/77 (13)	3/74 (4.1)	0.17	0.04–0.67	**0.011**
**Serum Creatinine (μmol/L)**	132.6 (80.4–231.6)	95.5 (63.6–130.8)	0.67	0.51–0.89	**0.006**
**Serum Sodium (mmol/L)**	141 (137–146)	139 (136–143)	0.96	0.93–1	0.063
**Serum Potassium (mmol/L)**	4.3 (3.8–5)	4–3 (3.7–4.9)	0.91	0.68–1.22	0.54
**Serum Bilirubin (μmol/L)**	13.5 (8.7–25)	8.9 (6.2–16.9)	0.72	0.52–0.99	**0.045**
**Serum Glucose (mmol/L)**	7.94 (5.33–11.32)	7.94 (6.48–10.45)	1	0.998–1.004	0.623
**Serum Albumin (g/L)**	29.5±7.3	32±7.5	1.64	1.05–2.56	**0.031**
**Predictive Scores**					
**APACHE II**	28 (23–34)	23 (18–29)	0.91	0.87–0.96	**<0.001**
**APACHE III**	109 (87.25–131.75)	87 (61–99)	0.97	0.96–0.98	**<0.001**
**APACHE IV**	99.5 (79.25–121)	76 (58–91)	0.97	0.96–0.98	**<0.001**
**SAPS II**	66.5 (53–78–75)	49.5 (37.75–62.25)	0.94	0.92–0.97	**<0.001**
**SAPS III**	85 (76–94)	70 (62–84)	0.96	0.93–0.98	**<0.001**
**SOFA**	9 (7.25–12)	6 (3–9)	0.77	0.69–0.86	**<0.001**
**MPM II Day0 mortality (%)**	73 (43.3–88)	41–8 (21.2–68.8)	0.97	0.96–0.99	**<0.001**
**MPM III Day0 mortality (%)**	77.3 (49.5–91.2)	39.5 (15.6–80.6)	0.98	0.97–0.99	**<0.001**

Univariate analysis.

*Some data is missing because some patients did not stay in the hospital long enough to collect information regarding their family status and complete medical history from their next of kin. Categorical variables are presented as n/N (%), continuous variables as mean ± SD or median [25th–75th percentile]. bpm: beats per minute, GCS: Glasgow Coma Scale, Dpt: Department, ICU: Intensive Care Unit, MAP: Mean arterial pressure, RR: respiratory rate, APACHE: Acute Physiology Assessment and Chronic Health Evaluation, SAPS: Simplified Acute Physiology Score, SOFA: Sequential Organ Failure Assessment, MPM: Mortality Prediction Model.

**Table 4:** Multivariate binary logistic regression analysis for the prediction of ICU transfer.

Risk factor	aOR	95%CI	P
**Female gender**	1.05	0.43–2.56	0.914
**Age**	1.01	0.98–1.05	0.434
**Charlson score**	0.75	0.6–0.94	**0.012**
**Main Indication**			
**Respiratory**	ref	ref	Ref
**Neurological**	0.26	0.09–0.82	**0.021**
**Cardiac arrest**	0.13	0.03–0.46	**0.002**
**Infection**			
**No infection**	ref	ref	Ref
**Community**	1.04	0.3–3.59	0.952
**Nosocomial**	0.98	0.29–3.35	0.978
**GCS**	1.17	1.03–1.34	**0.02**
**Platelet count (x 10^9^/L)**	1.01	1–1.02	**<0.001**
**Serum Creatinine (μmol/L)**	0.78	0.51–1.19	0.248
**Serum Bilirubin (μmol/L)**	0.61	0.36–1.04	0.072
**Serum Albumin (g/L)**	1.84	0.9–3.78	0.097
**SOFA**	1.08	0.87–1.34	0.477

Last Block of a hierarchical model available in the Supplementary material. **a**OR: adjusted odds ratio. CI: Confidence interval. GCS: Glasgow Coma Scale, SOFA: Sequential Organ Failure Assessment.

### Survival comparison based on ICU transfer

In the subgroup of patients transferred to the ICU, the median time from intubation to transfer was different between survivors and non-survivors [0 days (0–1), range = 1 vs. 1 day (0–1), range = 7, p = 0.07], albeit marginally without statistical significance. There was, however, a statistically significant difference in in-hospital [64.9% (48/74) vs. 97.4% (75/77), p<0.001] and 90-day mortality [69.9% (51/73) vs. 98.7% (76/77), p<0.001] when comparing, the transferred subgroup to the non-transferred one in favor of the first. The difference in survival between the two subgroups is illustrated in the Kaplan Meier survival analysis (Fig 3).

### Predictors of survival in the ICU

Given that ICU transfer had a decisive impact on survival, since mortality was almost 100% in the non-ICU transferred group (76/77), we investigated the predictors of mortality exclusively in the ICU-transferred group (Table 5 and S6 Table). The SOFA score showed the best diagnostic accuracy with an AUROC of 0.818 and 0.855 for in-hospital and 90-day mortality, respectively. A baseline SOFA score ≤8 was 100% sensitive (and 58% specific) in identifying the twenty-two 90-day survivors in the ICU-transferred population. When applied to the non-ICU transferred population, this cut-off identified 26 individuals out of whom only one survived (SOFA score = 5). On the flip side, there were 19 patients with a SOFA score >8 who were transferred to an ICU and did not survive and 51 patients with a baseline SOFA score >8 who were not transferred and all of whom died in the wards. Using as the optimal cut-off SOFA value of 5, sensitivity was 83% and 84% and specificity 62.5% and 75%, for in-hospital and 90-day mortality, respectively, for those transferred to the ICU.

**Table 5:** Demographic, clinical, and laboratory characteristics at the time of their intubation of survivors vs. non-survivors over the 90-day follow-up amongst the population that was transferred to the ICU during the hospital stay.

	90-day survival	90-day mortality	HR	95%CI	p
	(N = 22)	(N = 51)			
**Patient Characteristics**					
**Female gender**	12/22 (54.5)	22/51 (69.9)	0.91	0.51–1.6	0.733
**Age**	58.5 (40.8–71.3)	72 (61–80)	1.021	1–1.04	**0.022**
With spouse*	10/20 (50)	33/47 (70.1)	2.19	1-15-4.19	**0.017**
With offspring*	15/21 (71.4)	39/51 (76.5)	1.34	0.682.62	0.398
**Charlson score**	2 (1–6)	6 (3–7)	1.14	1.05–1.24	**0.003**
**Intubation Information**					
**Main Indication**					
**Respiratory**	12/22 (54.5)	31/51 (60.8)	ref	ref	ref
**Neurological**	9/22 (40.9)	12/51 (23.5)	0.75	0.37–1.49	0.406
**Cardiac arrest**	1/22 (4.5)	8/51 (15.7)	1.21	0.55–2.66	0.634
**Location**					
**Emergency Dpt**	12/22 (54.5)	8/51 (15.7)	ref	ref	ref
**Ward**	10/22 (45.5)	38/51 (74.5)	2.51	1.11–5.65	**0.027**
**Other**	0/22 (0)	5/22 (9.8)	5.79	1.8–18.68	**0.003**
**On weekdays**	14/22 (63.6)	37/22 (72.5)	1.31	0.7–2.43	0.403
**Hospital status**					
**Normal night**	6/22 (27.3)	18/51 (35.3)	ref	ref	ref
**On-call night**	14/22 (63.6)	17/51 (33.3)	0.6	0.31–1.19	0.143
**Morning shift**	2/22 (9.1)	16/61 (31.4)	1.34	0.66–2.7	0.415
**Emergency indication**	9/22 (40.9)	20/51 (39.2)	1.23	0.69–2.19	0.486
**Circulatory support**	0/22 (0)	13/51 (25.5)	4.02	2.07–7.8	**<0.001**
**Infection**					
**No infection**	17/22 (77.3)	32/51 (62.7)	ref	ref	ref
**Community**	5/22 (22.7)	9/51 (17.6)	0.9	0.43–1.9	0.782
**Nosocomial**	0/22 (0)	10/51 (19.6)	3.54	1.64–7.63	**0.001**
**Septic Shock**	0/22 (0)	10/51 (19.6)	2.85	1.36–5.97	**0.005**
**Vital signs and laboratory values immediately before intubation**					
**Heart rate (bpm)**	106.5 (91.8–116.3)	93 (85–113.8)	1	0.99–1.01	0.993
**MAP (mmHg)**	90 (86.7–97.5)	81.7 (61.3–97.5)	0.99	0.98–0.99	**0.009**
**Temperature (°C)**	36.6 (36.5–37.3)	36.6 (36.2–37.1)	0.89	0.63–1.26	0.503
**RR (/min)**	30 (14.5–40)	29 (17.8–35)	0.99	0.98–1.02	0.782
**Blood pH**	7.33 (7.21–7.4)	7.23 (7.15–7.4)	0.59	0.14–2.57	0.483
**PO2/FiO2**	189 (95.8–330)	113.5 (73–220.5)	0.99	0.99–1	0.055
**GCS**	10 (3.8–15)	11 (5–14)	0.99	0.94–1.06	0.897
**Henatocrit (%)**	35.8 (34.4–38.7)	32.6 (28–38.9)	0.944	0.91–0.98	**0.006**
**White blood cells (x 109/L)**	10.6 (5.8–15.7)	13.7 (7.4–18.9)	1.01	1–1.03	0.055
**Neutropenia**	0/22 (0)	1/51 (2)	11.2	1.35–93.1	**0.025**
**Platelet count (x 109/L)**	216 (190–268.3)	191 (108–309)	0.99	0.99–1	0.093
**>150**	20/22 (90.9)	33/51 (64.7)	rerf	ref	ref
**100–149**	2/22 (9.1)	6/51 (11.8)	1.45	0.6–3.5	0.405
**50–99**	0/22 (0)	5/51 (9.8)	5.5	2.04–14.8	**0.001**
**20–49**	0/22 (0)	4/51 (7.8)	3.03	1.07–8.63	**0.038**
**<20**	0/22 (0)	3/51 (5.9)	18.67	4.67–74.66	**<0.001**
**Serum Creatinine (μmol/L)**	69 (52.2–98.1)	110.5 (65.4–149.4)	1.43	1.15–1.78	**0.002**
**Serum Sodium (mmol/L)**	139 (135–142)	139 (136–146)	1	0.97–1.04	0.851
**Serum Potassium (mmol/L)**	4.3 (3.7–5.1)	4.3 (3.8–4.9)	0.944	0.67–1.33	0.743
**Serum Bilirubin (μmol/L)**	8.2 (5–14.7)	9.1 (6.8–26.3)	1.47	1.15–1.87	**0.002**
**Serum Glucose (mmol/L)**	7.83 (6.41–10)	8 (6.5–10.8)	1.003	1–1.005	0.064
**Serum Albumin (g/L)**	35.2 ± 6.3	31 ± 7.8	0.68	0.46–1.03	0.067
**Predictive Scores**					
**APACHE II**	14.5 (11.3–20)	24.5 (21–31_	1.1	1.06–1.14	**<0.001**
**APACHE III**	57.5 (30.8–79)	91.5 (76.8–117.3)	1.02	1.01–1.03	**<0.001**
**APACHE IV**	53 (34.8–72.8)	83 (67.8–98.3)	1.02	1.01–1.03	**<0.001**
**SAPS II**	34.5 (29.8–47)	53 (43.8–64.5)	1.04	1.02–1.05	**<0.001**
**SAPS III**	59 (46.8–67.3)	75.5 (67–86.3)	1.07	1.04–1.09	**<0.001**
**SOFA**	3 (2–4.8)	7.5 (5–11_	1.31	1.02–1.4	**<0.001**
**MPM II Day 0 mortality (%)**	22.8 (14.5–32.8)	53.2 (28.2–73.1)	1.03	1.02–1.04	**<0.001**
**MPM III Day 0 mortality (%)**	17.2 (9.4–30.8)	58.3 (23–86.1)	1.02	1.01–1.03	**<0.001**
**Transfer >1 day after intubation**	22/22 (100)	40/51 (78.4)	2.69	1.31–5.5	**0.007**

Univariate analysis using Cox proportional hazard regression. One patient was lost during follow-up and thus 73 patients were included out of the 74 that were transferred to ICUs.

*Some data is missing because some patients did not stay in our hospital long enough to collect information regarding their family status. Categorical variables presented as n/N (%), continuous variables presented as mean ± SD or median [25th–75th percentile]. Ref: reference, GCS: Glasgow Coma Scale, Dpt: Department, ICU: Intensive Care Unit, bpm: beats per minute, MAP: Mean arterial pressure, RR: respiratory rate, APACHE: Acute Physiology Assessment and Chronic Health Evaluation, SAPS: Simplified Acute Physiology Score, SOFA: Sequential Organ Failure Assessment, MPM: Mortality Prediction Model.

## Discussion

In our study we present a grim picture for patients who receive mechanical ventilation in medical wards. Total in-hospital mortality exceeded 80%, while 90-day mortality was somewhat higher, reaching 85%. Half of the intubated patients were, at some point, transferred to an ICU, and out of those, one third survived. On the other hand, mortality was almost 100% in those remaining in the wards.

Physicians working in the IM wards have limited experience with critical care. Almost all the resident doctors receive training in cardiopulmonary resuscitation in collaboration with the Hellenic Society of Cardiopulmonary Resuscitation upon starting their residency. During their 4th or 5th year they also receive a training rotation in an ICU for 3 months. Likewise, attending physicians have limited exposure to ICU settings. The critical care physicians from the ICU contribute to any intubated patient’s care via consultations on ventilation parameters and fluid management when requested by the medical team in charge. Each medical ward has a single vital-signs-monitor that is usually employed in the treatment of such patients. When the monitor is not available, residents record vital signs, usually several times per day, but only once or twice in the night. Mobile radiology services are available on-demand and if there is a need for dialysis the patients need to be transferred for the duration to the dialysis unit. Arterial lines are rarely employed in the wards, but blood is drawn for gas analysis every day.

Although non-ICU transferred patients had an overall less favorable prognosis at baseline, almost a third of them had a SOFA prognostic score falling within the range identifying ICU-transferred survivors with a sensitivity of 100%. The SOFA cut-off score of 8, bearing a sensitivity of 100% to predict survival, means that no patient with a score above this value would survive. In our study, we identified 25 patients in the non-transferred group with SOFA scores below the ICU survival threshold who were not transferred (thus received sub-optimal care) and eventually died in the medical wards (one additional patient survived) and 19 patients who were above this threshold, who were transferred to the ICU, yet still did not survive, indicating that they received care that could be termed as futile.

We opted, at first, to perform our analysis by investigating only factors that were present when the decision to intubate was taken, since these parameters may serve as a guide for clinicians to identify those patients for whom mechanical ventilation might be futile. The factors which independently predicted in-hospital mortality in our multivariable model were the Charlson and SOFA scores, cardiac arrest, and a low platelet count. When transfer to an ICU was introduced in the last hierarchical step of the model, it was the only factor retaining statistical significance, together with the SOFA score. This finding should, however, be interpreted with caution. Undoubtedly, a subsequent (to the intubation) patient-transfer to an ICU is beneficial for survival. Nevertheless, this parameter is, by default, associated with survival since some patients died before having the chance to be transferred, while, others, might have survived without being transferred. Most importantly, the decision to transfer is strongly related to the patient’s clinical condition and prognosis. This is clearly reflected in the multivariate regression model with ICU transfer as the dependent variable (Table 2), showing that Charlson score, cardiac arrest, and platelet count are again significant predictors. This mirror image indicates that mortality predictors influence decisions by the ICU’s staff regarding who will get transferred, given the limited availability of ICU beds. In addition, the fact that GCS and the presence of a neurological condition as an indication for mechanical ventilation, also negatively predicted ICU transfer (Table 2), could indicate a hesitation to transfer stroke patients to the ICU given the poor prognosis they carry for eventual weaning from the ventilator and rehabilitation. On the other hand, the fact that the SOFA score does not display statistical significance as a predictor for ICU transfer, could reflect that the ventilation conditions using a portable ventilator in the IM department are sub-optimal compared to the mechanical ventilators of the ICU, enabling better outcomes for patients with respiratory insufficiency, thus the SOFA scores themselves are not of immediate concern to ICU clinicians. With these in mind, the 26 individuals who were not transferred to an ICU albeit having good chances of survival vs. the 19 that perhaps received futile care in the ICU, is something that physicians ought to reflect upon.

In recent international cohorts, in-hospital mortality for patients that received mechanical ventilation in an ICU has ranged from 23.9 to 39.2% [20, 21], however both medical and surgical patients are included in these studies and there is a selection bias, since only patients admitted to the ICU in the first place were enrolled. On the other hand, the high mortality in our study prompts the question of whether some of the patients included are receiving futile treatment.

Similar studies have been conducted in Israel, where due to religious reasons, the majority of patients receive mechanical ventilation regardless of their prognosis [22, 23]. Hersch et al. reported an overall mortality of 80% for patients ventilated in the medical wards compared to 62% in the ICU. Lieberman et al. found statistical significance comparing mortality of elderly patients in the ICU and outside (53% vs. 68.2%, p<0.001), however this finding did not maintain significance in multivariate analysis. Of note is also the fact that patients ventilated in the wards were older and frailer, indicating the selection bias applied by the ICUs in admitting patients [24]. The importance of ICU admission for survival has been also demonstrated by Sprung et al., in whose study, hospital mortality was only 14% for patients admitted to an ICU vs. 36% for those with delayed admission and 46% for those not admitted [25]. Amongst patients older than 75, Pintado et al. showed 1-year mortality of 73.7% for those not admitted to ICU vs. 42.5% for those admitted [26]. In a study from Hong Kong, Tang et al. also reported an overall hospital mortality of 89.1% (673/755), with a worse prognosis following cardiac arrest and a better prognosis for COPD (chronic obstructive pulmonary disease) patients. Of note, only 28.7% (217/755) of the participants were referred to ICUs for transfer and were refused admission either due to lack of beds (60/217) or due to them being deemed too severely ill to have any benefit from transferring to the ICU (157/217). Mortality in the group was 93% [27]. Finally, in a study from Thailand, researchers found that only the APACHE II score was correlated to hospital mortality in multiple regression analysis using a cut-off value of 22 and with an overall mortality of 68.8% [28].

Our study provides a comprehensive evaluation of parameters that can predict patient mortality both during hospitalization and for 90 days post-intubation. To our knowledge it is the first attempt to assess the predictive value of ICU prognostic scores in determining these outcomes for patients who receive mechanical ventilation in medical wards. In particular, comparing the different predictive scores, we found that albeit all scores predicted mortality with statistical significance, the SOFA score had the largest AUROC while, at the same time, it is by far the simplest to use, having the fewest parameters and being easy to calculate. This may allow physicians to make decisions regarding intubations even in emergency situations with limited time and available data.

There are, of course limitations to our study. First, we describe the experience of our hospital, one of the largest in Greece and a good example of hospital standards of operating procedures in our country. This is not representative however of other countries, although we believe that the situations described may compare to middle- and lower-income settings. Nonetheless, validation of these findings in different settings is warranted to generalize conclusions. Second, it is possible that certain parameters that would affect outcomes were not measured. Our selection was based on existing literature and based on our study’s aims. A third issue is, that, there exists a subpopulation of patients that would have been transferred to an ICU had there been given time for it to happen. As we demonstrated though, the majority of patients were transferred the same day or the next, so this subpopulation is probably small. Finally, the sample size is relatively small, which is related to the fact that this was a single-hospital study and the recruitment was limited.

## Conclusions

In conclusion, mortality in patients receiving mechanical ventilation in medical wards is very high, although somewhat mitigated if the patient is eventually transferred to an ICU. Lower SOFA and Charlson scores predict a higher chance of survival, but further studies are needed to identify cases for which intubation and mechanical ventilation, under these circumstances, might be futile. In any case, although the majority of non-ICU transferred patients have an unfavorable prognosis, a proportion of them might have increased chances to survive if transferred. Lack of ICU beds means that the selection of patients receiving mechanical ventilation needs to be reviewed as patients with the worse clinical picture and underlying conditions are less likely to be transferred to an ICU and survive hospitalization.

## Supporting information

S1 Table Demographic, clinical, and laboratory characteristics at the time of intubation of survivors vs. non-survivors during the hospital stay. Univariate analysis using Cox proportional hazard regression.**Some data is missing because some patients did not stay in our hospital long enough to collect information regarding their family status. Categorical variables presented as n/N (%), continuous variables presented as mean ± SD or median [25th–75th percentile]. Ref: reference, GCS: Glasgow Coma Scale, Dpt: Department, ICU: Intensive Care Unit, MAP: Mean arterial pressure, RR: respiratory rate, APACHE: Acute Physiology Assessment and Chronic Health Evaluation, SAPS: Simplified Acute Physiology Score, SOFA: Sequential Organ Failure Assessment, MPM: Mortality Prediction Model.
(DOCX)

S2 Table Demographic, clinical, and laboratory characteristics at the time of intubation of survivors vs. non-survivors over the 90-day follow-up. Univariate analysis using Cox proportional hazard regression. *One patient was lost during follow-up and thus N = 130 and not 131. **Some data is missing because some patients did not stay in our hospital long enough to collect information regarding their family status. Categorical variables presented as n/N (%), continuous variables presented as mean ± SD or median [25th–75th percentile]. Ref: reference, GCS: Glasgow Coma Scale, Dpt: Department, ICU: Intensive Care Unit, MAP: Mean arterial pressure, RR: respiratory rate, APACHE: Acute Physiology Assessment and Chronic Health Evaluation, SAPS: Simplified Acute Physiology Score, SOFA: Sequential Organ Failure Assessment, MPM: Mortality Prediction Model.
(DOCX)

S3 Table Multivariate Cox proportional hazards regression analysis for the prediction of in-hospital mortality. Complete hierarchical model. aHR: Adjusted Hazard Ratio. CI: Confidence interval. MAP: Mean arterial pressure. SOFA: Sequential Organ Failure Assessment, ICU: Intensive Care Unit.
(DOCX)

S4 Table Multivariate Cox proportional hazards regression analysis for the prediction of 90-day mortality. Complete hierarchical model. aHR: Adjusted Hazard Ratio. CI: Confidence interval. MAP: Mean arterial pressure. SOFA: Sequential Organ Failure Assessment, ICU: Intensive Care Unit.
(DOCX)

S5 Table Multivariate binary logistic regression analysis for the prediction of ICU transfer. Complete hierarchical model. **a**OR: adjusted odds ratio. CI: Confidence interval. GCS: Glasgow Coma Scale, SOFA: Sequential Organ Failure Assessment.
(DOCX)

S6 Table Multivariate Cox regression analysis for the mortality of patients transferred to ICU departments. Complete hierarchical model. aHR: adjusted hazards ratio. CI: Confidence interval. MAP: mean arterial pressure, SOFA: Sequential Organ Failure Assessment.
(DOCX)
