# Supplementary material for: Survival predictors after intubation in medical wards: A prospective study in 151 patients
Source: PLoS One. 2020 Jun 1;15(6):e0234181. doi: 10.1371/journal.pone.0234181 (PMC7263577; doi:10.1371/journal.pone.0234181)
Supplement: S1 Table — Univariate analysis using Cox proportional hazard regression.**Some data is missing because some patients did not stay in our hospital long enough to collect information regarding their family status. Categorical variables presented as n/N (%), continuous variables presented as mean ± SD or median [25th–75th percentile]. Ref: reference, GCS: Glasgow Coma Scale, Dpt: Department, ICU: Intensive Care Unit, MAP: Mean arterial pressure, RR: respiratory rate, APACHE: Acute Physiology Assessment and Chronic Health Evaluation, SAPS: Simplified Acute Physiology Score, SOFA: Sequential Organ Failure Assessment, MPM: Mortality Prediction Model. (DOCX) [file pone.0234181.s001.docx]

**Supporting Material**

**S1 Table: Demographic, clinical and laboratory characteristics at the time of intubation of survivors vs. non-survivors during hospital stay.**

|  | In-hospital survival | In-hospital mortality | HR | 95%CI | p | |
| --- | --- | --- | --- | --- | --- | --- |
|  | **(N=28)** | **(N=123)** |  |  |  |  |
| Patient Characteristics | | | | | |  |
| Female gender | 15/28 (53.6) | 59/123(48) | 0.79 | 0.55-1.31 | 0.788 | |
| Age | 62.5 (41,3 - 79.8) | 72 (61 - 80) | 1.01 | 0.99-1.02 | 0.146 | |
| With spouse** | 10/25 (40) | 73/117 (62.4) | 1.45 | 0.99-2.12 | 0.056 | |
| With offspring** | 19/27 (70.4) | 90/121 (74.4) | 1.23 | 0.81-1.85 | 0.335 | |
| Charlson score | 4 (1 - 6) | 6 (4 - 7) | 1.09 | 1.02-1.16 | **0.008** | |
| Intubation Information | | | | | |  |
| Main Indication |  |  |  |  |  | |
| Respiratory | 15/28(53.6) | 48/123 (39) | ref | ref | ref | |
| Neurological | 11/28 (39.3) | 43/123 (35) | 1.68 | 1.1-2.59 | **0.018** | |
| Cardiac arrest | 2/28 (7.1) | 32/123 (26) | 2.36 | 1.49-3.76 | **<0.001** | |
| Location |  |  |  |  |  | |
| Emergency Dpt | 15/28 (53.6) | 24/123 (19.5) | ref | ref | ref | |
| Ward | 13/28 (46.4) | 91/123 (74) | 1.21 | 0.77-1.93 | 0.403 | |
| Other | 0/28 (0) | 9/123 (7.3) | 1.92 | 0.89-4.16 | 0.097 | |
| On weekdays | 18/28 (64.3) | 80/123 (65) | 1.14 | 0.78-1.65 | 0.509 | |
| Hospital status |  |  |  |  |  | |
| Normal night | 8/28 ( 28.6) | 44/123(35.8) | ref | ref | ref | |
| On call night | 17/28(60.7) | 46/123 (37.4) | 1.05 | 0.69-1.59 | 0.822 | |
| Morning shift | 3/28 (10.7) | 33/123 (26.8) | 1.27 | 0.8-2.02 | 0.303 | |
| Emergency indication | 18/28 (64.3) | 82/123 (66.7) | 1.2 | 0.82-1.76 | 0.344 | |
| Circulatory support | 0/28 (0) | 35/123 (28.5) | 2.19 | 1.46-3.29 | **<0.001** | |
| Infection |  |  |  |  |  | |
| No infection | 24/28 (85.7) | 67/123 (54.5) | ref | ref | ref | |
| Community | 4/28 (14.3) | 26/123 (21.1) | 1.01 | 0.64-1.6 | 0.974 | |
| Nosocomial | 0/28 (0) | 30/123 (24.4) | 2.33 | 1.49-3.63 | **<0.001** | |
| Septic Shock | 0/28 (0) | 21/123 (17.1) | 1.73 | 1.06-2.8 | **0.027** | |
| Vital signs and laboratory values immediately before intubation | | | | | |  |
| Heart rate (/min) | 102.5 (87 - 113.8) | 99.5 (85 - 116.3) | 1 | 0.96-1.01 | 0.687 | |
| MAP (mmHg) | 90 (86.7 - 99.2) | 78 (58.2 - 96.7) | 0.99 | 0.98-1 | **0.001** | |
| Temperature (°C) | 36.6 (36.3 - 37) | 36.6 (36.2 - 37.8) | 1.05 | 0.9-1.23 | 0.504 | |
| RR (/min) | 20.5 (12.3 - 35) | 28.5 (18 - 35) | 1 | 0.99-1.02 | 0.827 | |
| Blood pH | 7.31 (7.21 - 7.39) | 7.29 (7.15 - 7.41) | 0.67 | 0.24-1.88 | 0.449 | |
| PΟ_2_/FiO_2_ | 191.5 (95.8 - 336.8) | 116 (79.5 - 232.5) | 1 | 0.99-1 | 0.946 | |
| GCS | 8 (3.3 - 15) | 8 (3 - 14) | 0.98 | 0.94-1.01 | 0.187 | |
| Henatocrit (%) | 35.9 (34.2 - 39.5) | 31 (26 - 37.6) | 1 | 0.99-1.01 | 0.981 | |
| White blood cells (x 10^9^/L) | 11.05 (6.83 - 17.97) | 11.16 (5.27 - 18.18) | 1 | 0.99-1.01 | 0.37 | |
| Neutropenia | 0/28 (0) | 5/123 (4.1) | 3.3 | 1.31-8.24 | **0.011** | |
| Platelet count (x 10^9^/L) | 222.5 (184 - 286.3) | 139 (49.8 - 233.3) | 0.996 | 0.994-0.997 | **<0.001** | |
| >150 | 26/28 (92.9) | 60/123 (48.8) | ref | ref | ref | |
| 100-149 | 2/28 (7.1) | 18/123 (14.6) | 1.73 | 1.02-2.95 | **0.044** | |
| 50-99 | 0/28 (0) | 15/123 (12.2) | 2.69 | 1.5-4.82 | **0.001** | |
| 20-49 | 0/28 (0) | 17/123 (13.8) | 3.13 | 1.8-5.46 | **<0.001** | |
| <20 | 0/28 (0) | 13/123 (10.6) | 4.20 | 2.2-7.44 | **<0.001** | |
| Serum Creatinine (mmol/L) | 74.3 (54.8 – 101.7) | 123.8 (76.9 - 214) | 1.07 | 1.01-1.14 | **0.03** | |
| Serum Sodium (mmol/L) | 139.5 (137 - 142.8) | 140 (136 - 146) | 1.01 | 0.98-1.03 | 0.645 | |
| Serum Potassium (mmol/L) | 4.3 (3.7 - 4.9) | 4.3 (3.8 - 5) | 1.01 | 0.86-1.19 | 0.9 | |
| Serum Bilirubin (μmol/L) | 8.2 (4.8 – 13.9) | 12.1 (7.9 – 25.5) | 1.27 | 1.12-1.44 | **<0.001** | |
| Serum Glucose (mmol/L) | 7.6 (6.4 - 10) | 8 (5.6 – 11.5) | 1 | 0.999-1.002 | 0.524 | |
| Serum Albumin (g/L) | 34.9 ± 6 | 29.9 ± 7.6 | 0.83 | 0.65-1.06 | 0.134 | |
| Predictive Scores | | | | | |  |
| APACHE II | 19 (12.8 - 24.5) | 27 (22 - 33) | 1.06 | 1.04-1.08 | **<0.001** | |
| APACHE III | 67.5 (49 - 90.3) | 101 (85 - 127) | 1.02 | 1.01-1.02 | **<0.001** | |
| APACHE IV | 68 (39.3 - 84) | 92 (74.5 - 115.5) | 1.01 | 1.01-1.02 | **<0.001** | |
| SAPS II | 38 (32 - 54) | 61 (49 - 73.5) | 1.03 | 1.02-1.04 | **<0.001** | |
| SAPS III | 63 (53.5 - 70.8) | 82 (70.5 - 92) | 1.04 | 1.03-1.05 | **<0.001** | |
| SOFA | 4 (2 - 6) | 9 (7 - 11) | 1.2 | 1.15-1.27 | **<0.001** | |
| MPM II Day 0 mortality (%) | 28.4 (16.6 - 59.4) | 66 (40.7 - 85.9) | 1.02 | 1.01-1.03 | **<0.001** | |
| MPM III Day 0 mortality (%) | 24.4 (11.2 - 52.7) | 70.5 (41.7 - 88.8) | 1.02 | 1.01-1.02 | **<0.001** | |
| Transfer to ICU | 26/28 (92.9) | 48/123 (49) | 0.15 | 0.09-0.24 | <0.001 | |

Univariate analysis using Cox proportional hazard regression.**Some data is missing, because some patients did not stay in our hospital long enough to collect information regarding their family status. Categorical variables presented as n/N (%), continuous variables presented as mean ± SD or median [25th - 75th percentile]. Ref: reference, GCS: Glasgow Coma Scale, Dpt: Department, ICU: Intensive Care Unit, MAP: Mean arterial pressure, RR: respiratory rate, APACHE: Acute Physiology Assessment and Chronic Health Evaluation, SAPS: Simplified Acute Physiology Score, SOFA: Sequential Organ Failure Assessment, MPM: Mortality Prediction Model.
