# Supplementary material for: Survival predictors after intubation in medical wards: A prospective study in 151 patients
Source: PLoS One. 2020 Jun 1;15(6):e0234181. doi: 10.1371/journal.pone.0234181 (PMC7263577; doi:10.1371/journal.pone.0234181)
Supplement: S2 Table — Univariate analysis using Cox proportional hazard regression. *One patient was lost during follow-up and thus N = 130 and not 131. **Some data is missing because some patients did not stay in our hospital long enough to collect information regarding their family status. Categorical variables presented as n/N (%), continuous variables presented as mean ± SD or median [25th–75th percentile]. Ref: reference, GCS: Glasgow Coma Scale, Dpt: Department, ICU: Intensive Care Unit, MAP: Mean arterial pressure, RR: respiratory rate, APACHE: Acute Physiology Assessment and Chronic Health Evaluation, SAPS: Simplified Acute Physiology Score, SOFA: Sequential Organ Failure Assessment, MPM: Mortality Prediction Model. (DOCX) [file pone.0234181.s002.docx]

**Supporting Material**

**S2 Table: Demographic, clinical and laboratory characteristics at the time of intubation of survivors vs. non-survivors over the 90-day follow-up.**

|  | | 90-day survival | | 90-day mortality* | | HR | | 95%CI | | p | |  |
| --- | --- | --- | --- | --- | --- | --- | --- | --- | --- | --- | --- | --- |
|  |  | **(n=23)** | | **(n=127)** | |  |  |  |  |  |  |  |
| Patient Characteristics | | | | | | | | | | | |  |
| Female gender | | 12/23 (52.2) | | 61/127 (48) | | 0.91 | | 0.64-1.3 | | 0.599 | |  |
| Age | | 56 (40 - 71) | | 73 (61 - 81) | | 1.02 | | 1.01-1.03 | | **0.003** | |  |
| With spouse** | | 10/21 (47.6) | | 72/120 (60) | | 1.31 | | 0.91-1.9 | | 0.147 | |  |
| With offspring** | | 15/22 (68.2) | | 93/125 (74.4) | | 1.18 | | 0.79-1.78 | | 0.421 | |  |
| Charlson score | | 2 (1 - 6) | | 6 (4 - 7) | | 1.13 | | 1.07-1.2 | | **<0.001** | |  |
| Intubation Information | | | | | | | | | | | |  |
| Main Indication | |  | |  | |  | |  | |  | |  |
| Respiratory | | 12/23 (52.2) | | 50/127 (39.4) | | ref | | ref | | ref | |  |
| Neurological | | 10/23 (43.5) | | 44/127 (34.6) | | 1.26 | | 0.83-1.9 | | 0.28 | |  |
| Cardiac arrest | | 1/23 (4.3) | | 33/127 (26) | | 2.04 | | 1.3-3.19 | | **0.002** | |  |
| Location | |  | |  | |  | |  | |  | |  |
| Emergency Dpt | | 13/23 (56.5) | | 26/127 (20.5) | | ref | | ref | | ref | |  |
| Ward | | 10/23 (43.5) | | 93/127 (73.2) | | 1.69 | | 1.08-2.67 | | **0.023** | |  |
| Other | | 0/23 (0) | | 9/127 (7.1) | | 2.67 | | 1.23-5.79 | | **0.013** | |  |
| On weekdays | | 15/23 (65.2) | | 82/127 (64.6) | | 1.05 | | 0.73-1.51 | | 0.802 | |  |
| Hospital status | |  | |  | |  | |  | |  | |  |
| Normal night | | 6/23 (26.1) | | 45/127 (35.4) | | ref | | ref | | ref | |  |
| On call night | | 15/23 (65.2) | | 48/127 (37.8) | | 0.8 | | 0.53-1.21 | | 0.298 | |  |
| Morning shift | | 2/23 (8.7) | | 34/127 (26.8) | | 1.24 | | 0.79-1.95 | | 0.357 | |  |
| Emergency indication | | 14/23 (60.9) | | 85/127 (66.9) | | 1.27 | | 0.87-1.85 | | 0.213 | |  |
| Circulatory support | | 0/23 (0) | | 35/127 (27.6) | | 2.37 | | 1.58-3.56 | | **<0.001** | |  |
| Infection | |  | |  | |  | |  | |  | |  |
| No infection | | 18/23 (78.3) | | 72/127 (56.7) | | ref | | ref | | ref | |  |
| Community | | 5/23 (21.7) | | 25/127 (19.7) | | 1.01 | | 0.64-1.59 | | 0.983 | |  |
| Nosocomial | | 0/23 (0) | | 30/127 (23.6) | | 2.48 | | 1.58-3.89 | | **<0.001** | |  |
| Septic Shock | | 0/23 (0) | | 21/127 (16.5) | | 1.88 | | 1.16-3.05 | | **0.011** | |  |
| Vital signs and laboratory values immediately before intubation | | | | | | | | | | | |  |
| Heart rate (/min) | | 105.8 (86 - 115) | | 96.5 (85 - 115.3) | | 1 | | 0.99-1.01 | | 0.879 | |  |
| MAP (mmHg) | | 90 (86.7 - 96.7) | | 80 (59.6 - 96.9) | | 0.99 | | 0.98-1 | | **<0.001** | |  |
| Temperature (°C) | | 36.6 (36.4 - 37.3) | | 36.6 (36.2 - 37.8) | | 1.09 | | 0.93-1.26 | | 0.291 | |  |
| RR (/min) | | 30 (13 - 40) | | 28 (17.8 - 35) | | 1 | | 0.99-1.02 | | 0.676 | |  |
| Blood pH | | 7.32 (7.22 - 7.4) | | 7.28 (7.15 - 7.41) | | 0.74 | | 0.28-1.95 | | 0.54 | |  |
| PΟ_2_/FiO_2_ | | 211 (98.5 - 338.5) | | 116 (79.5 - 244.1) | | 1 | | 0.997-1 | | 0.067 | |  |
| GCS | | 10 (3 - 15) | | 8 (3 - 14) | | 0.98 | | 0.94-1.02 | | 0.266 | |  |
| Henatocrit (%) | | 35.8 (34.4 - 38.3) | | 31.4 (26.1 - 38.5) | | 0.99 | | 0.98-1.01 | | 0.769 | |  |
| White blood cells (x 10^9^/L) | | 10.36 (5.86 - 15.59) | | 11.4 (5.38 - 18.55) | | 1.01 | | 1-1.01 | | 0.194 | |  |
| Neutropenia | | 0/5 (0) | | 5/5 (100) | | 3.34 | | 1.34-8.38 | | **0.01** | |  |
| Platelet count (x 10^9^/L) | | 215 (181 - 265) | | 149.5 (59 - 235) | | 0.996 | | 0.994-0.998 | | **<0.001** | |  |
|  | **>150** | | 21/23 (91.3) | | 63/127 (49.6) | | **ref** | | **ref** | | ref | |
|  | **100-149** | | 2/23 (8.7) | | 18/127 (14.2) | | **1.83** | | **1.08-3.11** | | **0.025** | |
|  | **50-99** | | 0/23 (0) | | 15/127 (11.8) | | **2.93** | | **1.63-5.26** | | **<0.001** | |
|  | **20-49** | | 0/23 (0) | | 17/127 (13.4) | | **3.69** | | **2.12-6.42** | | **<0.001** | |
|  | **<20** | | 0/23 (0) | | 13/127 (10.3) | | **4.48** | | **2.37-8.47** | | **<0.001** | |
| Serum Creatinine (μmol/L) | | 70.7 (52.2 – 97.2) | | 123.8 (76.9 – 213.9) | | 1.09 | | 1.02-1.2 | | **0.008** | |  |
| Serum Sodium (mmol/L) | | 139 (137 - 142) | | 140 (136 - 146) | | 1.01 | | 0.99-1.04 | | 0.198 | |  |
| Serum Potassium (mmol/L) | | 4.3 (3.7 - 5) | | 4.3 (3.8 - 4.9) | | 1.03 | | 0.87-1.21 | | 0.766 | |  |
| Serum Bilirubin (μmol/L) | | 8.2 (4.8 – 14.7) | | 12 (7.5 – 25.5) | | 1.29 | | 1.14-1.46 | | **<0.001** | |  |
| Serum Glucose (mmol/L) | | 7.8 (6.3 - 10) | | 8 (5.6 - 11.1) | | 1 | | 0.99-1 | | 0.226 | |  |
| Serum Albumin (g/L) | | 35.4 ± 6.3 | | 30.0 ± 7.5 | | 0.72 | | 0.57-0.92 | | **0.009** | |  |
| Predictive Scores | | | | | | | | | |  | |  |
| APACHE II | | 15 (11.5 - 21) | | 27 (22 - 33) | | 1.07 | | 1.05-1.09 | | **<0.001** | |  |
| APACHE III | | 58 (41.5 - 84) | | 101 (85 - 126.5) | | 1.02 | | 1.01-1.02 | | **<0.001** | |  |
| APACHE IV | | 53 (35.5 - 75.5) | | 92 (75 - 115) | | 1.01 | | 1.01-1.02 | | **<0.001** | |  |
| SAPS II | | 36 (30.5) - 48.5) | | 61 (49 - 72.5) | | 1.03 | | 1.02-1.04 | | **<0.001** | |  |
| SAPS III | | 57 (48.5 - 66.5) | | 82 (70.5 - 92) | | 1.05 | | 1.04-1.06 | | **<0.001** | |  |
| SOFA | | 3 (2 - 5) | | 9 (6 - 11) | | 1.023 | | 1.17-1.29 | | **<0.001** | |  |
| MPM II Day 0 mortality (%) | | 24.4 (14.5 - 32.8) | | 64.5 (30.7 - 85.6) | | 1.02 | | 1.02-1.03 | | **<0.001** | |  |
| MPM III Day 0 mortality (%) | | 18.9 (9.4 - 30.4) | | 70.5 (39.2 - 88.8) | | 1.02 | | 1.01-1.02 | | **<0.001** | |  |
| Transfer to ICU | | 22/23 (95.7) | | 51/127 (40.2) | | 0.19 | | 0.13-0.29 | | **<0.001** | |  |

Univariate analysis using Cox proportional hazard regression. *One patient was lost during follow-up and thus N=130 and not 131. **Some data is missing, because some patients did not stay in our hospital long enough to collect information regarding their family status. Categorical variables presented as n/N (%), continuous variables presented as mean ± SD or median [25th - 75th percentile]. Ref: reference, GCS: Glasgow Coma Scale, Dpt: Department, ICU: Intensive Care Unit, MAP: Mean arterial pressure, RR: respiratory rate, APACHE: Acute Physiology Assessment and Chronic Health Evaluation, SAPS: Simplified Acute Physiology Score, SOFA: Sequential Organ Failure Assessment, MPM: Mortality Prediction Model.
