# Supplementary material for: Survival predictors after intubation in medical wards: A prospective study in 151 patients
Source: PLoS One. 2020 Jun 1;15(6):e0234181. doi: 10.1371/journal.pone.0234181 (PMC7263577; doi:10.1371/journal.pone.0234181)
Supplement: S3 Table — Complete hierarchical model. aHR: Adjusted Hazard Ratio. CI: Confidence interval. MAP: Mean arterial pressure. SOFA: Sequential Organ Failure Assessment, ICU: Intensive Care Unit. (DOCX) [file pone.0234181.s003.docx]

**Supporting Material**

**S3 Table: Multivariate Cox proportional hazards regression analysis for the prediction of in-hospital mortality.**

|  | Risk factor | aHR | 95%CI | p |
| --- | --- | --- | --- | --- |
| Block 1 | **Female gender** | 0.82 | 0.57-1.19 | 0.304 |
|  | **Age** | 1.01 | 0.99-1.01 | 0.321 |
| Block 2 | **Female gender** | 0.79 | 0.55-1.15 | 0.222 |
|  | **Age** | 0.99 | 0.98-1.01 | 0.857 |
|  | **Charlson** | 1.09 | 1.01-1.18 | 0.022 |
| Block 3 | **Female gender** | 0.81 | 0.56-1.18 | 0.268 |
|  | **Age** | 1 | 0.99-1.01 | 0.842 |
|  | **Charlson** | 1.06 | 0.98-1.15 | 0.137 |
|  | **Main Indication** |  |  |  |
|  | **Respiratory** | ref | ref | ref |
|  | **Neurological** | 1.79 | 1.14-2.81 | 0.012 |
|  | **Cardiac arrest** | 2.08 | 1.22-3.54 | 0.07 |
|  | **Location** |  |  |  |
|  | **Emergency Dpt** | ref | ref | ref |
|  | **Ward** | 1.28 | 0.78-2.12 | 0.331 |
|  | **Other** | 1.26 | 0.52-3.05 | 0.609 |
| Block 4 | **Female gender** | 0.77 | 0.52-1.15 | 0.208 |
|  | **Age** | 1 | 0.92-1.01 | 0.629 |
|  | **Charlson** | 1.06 | 0.98-1.15 | 0.148 |
|  | **Main Indication** |  |  |  |
|  | **Respiratory** | ref | ref | ref |
|  | **Neurological** | 1.83 | 1.16-2.91 | 0.01 |
|  | **Cardiac arrest** | 1.88 | 1.1-3.19 | 0.02 |
|  | **Location** |  |  |  |
|  | **Emergency Dpt** | ref | ref | ref |
|  | **Ward** | 1.06 | 0.63-1.78 | 0.83 |
|  | **Other** | 1.13 | 0.45-2.73 | 0.795 |
|  | **Circulatory Support** | 2.83 | 1.5-5.35 | 0.001 |
|  | **Infection** |  |  |  |
|  | **No infection** | ref | ref | ref |
|  | **Community** | 1.89 | 0.71-1.99 | 0.517 |
|  | **Nosocomial** | 2.4 | 1.35-4.3 | 0.003 |
|  | **Septic Shock** | 0.44 | 0.18-1.05 | 0.064 |
| Block 5 | **Female gender** | 0.86 | 0.6-1.32 | 0.544 |
|  | **Age** | 0.99 | 0.98-1 | 0.163 |
|  | **Charlson** | 1.17 | 1.07-1.28 | 0.001 |
|  | **Main Indication** |  |  |  |
|  | **Respiratory** | ref | ref | ref |
|  | **Neurological** | 1.84 | 1.14-2.96 | 0.013 |
|  | **Cardiac arrest** | 1.78 | 1-3.17 | 0.05 |
|  | **Location** |  |  |  |
|  | **Emergency Dpt** | ref | ref | ref |
|  | **Ward** | 0.92 | 0.52-1.62 | 0.763 |
|  | **Other** | 0.95 | 0.39-2.32 | 0.911 |
|  | **Circulatory Support** | 2.55 | 1.32-4.94 | 0.005 |
|  | **Infection** |  |  |  |
|  | **No infection** | ref | ref | ref |
|  | **Community** | 0.94 | 0.53-1.65 | 0.825 |
|  | **Nosocomial** | 1.72 | 0.9-3.3 | 0.105 |
|  | **Septic Shock** | 0.59 | 0.24-1.45 | 0.251 |
|  | **MAP (mmHg)** | 0.99 | 0.98-1 | 0.056 |
|  | **Neutropenia** | 1.71 | 0.56-5.22 | 0.347 |
|  | **Platelet count (x 10^9^/L)** | 0.995 | 0.99-1 | <0.001 |
|  | **Serum Creatinine (mg/dL)** | 1.02 | 0.93-1.12 | 0.687 |
|  | **Serum Bilirubin (mg/dL)** | 1.2 | 1.04-1.4 | 0.016 |
|  | **Serum Albumin (g/dL)** | 1.06 | 0.77-1.46 | 0.729 |
| Block 6 | **Female gender** | 0.92 | 0.62-1.37 | 0.675 |
|  | **Age** | 0.99 | 0.97-1 | 0.087 |
|  | **Charlson** | 1.14 | 1.04-1.25 | 0.006 |
|  | **Main Indication** |  |  |  |
|  | **Respiratory** | ref | ref | ref |
|  | **Neurological** | 1.5 | 0.91-2.48 | 0.114 |
|  | **Cardiac arrest** | 1.85 | 1.04-3.29 | 0.037 |
|  | **Location** |  |  |  |
|  | **Emergency Dpt** | ref | ref | ref |
|  | **Ward** | 0.85 | 0.48-1.51 | 0.572 |
|  | **Other** | 0.76 | 0.3-1.88 | 0.548 |
|  | **Circulatory Support** | 1.83 | 0.9-3.73 | 0.098 |
|  | **Infection** |  |  |  |
|  | **No infection** | ref | ref | ref |
|  | **Community** | 0.98 | 0.56-1.73 | 0.949 |
|  | **Nosocomial** | 1.83 | 0.96-3.48 | 0.066 |
|  | **Septic Shock** | 0.56 | 0.232-1.35 | 0.195 |
|  | **MAP (mmHg)** | 0.99 | 0.99-1 | 0.14 |
|  | **Neutropenia** | 1.49 | 0.48-4.58 | 0.487 |
|  | **Platelet count (x 10^9^/L)** | 0.99 | 0.99-1 | 0.004 |
|  | **Serum Creatinine (mg/dL)** | 0.95 | 0.85-1.07 | 0.387 |
|  | **Serum Bilirubin (mg/dL)** | 1.12 | 0.95-1.3 | 0.195 |
|  | **Serum Albumin (g/dL)** | 1.09 | 0.79-1.5 | 0.595 |
|  | **SOFA** | 1.14 | 1.09-1.27 | 0.013 |
| Block 7 | **Female gender** | 0.92 | 0.62-1.36 | 0.662 |
|  | **Age** | 0.99 | 0.98-1.01 | 0.506 |
|  | **Charlson** | 1.06 | 0.97-1.2 | 0.204 |
|  | **Main Indication** |  |  |  |
|  | **Respiratory** | ref | ref | ref |
|  | **Neurological** | 1.17 | 0.71-1.9 | 0.533 |
|  | **Cardiac arrest** | 1.4 | 0.78-2.5 | 0.26 |
|  | **Location** |  |  |  |
|  | **Emergency Dpt** | ref | ref | ref |
|  | **Ward** | 1 | 0.56-1.78 | 0.995 |
|  | **Other** | 1.26 | 0.49-3.23 | 0.63 |
|  | **Circulatory Support** | 1.33 | 0.66-2.7 | 0.431 |
|  | **Infection** |  |  |  |
|  | **No infection** | ref | ref | ref |
|  | **Community** | 0.8 | 0.45-1.44 | 0.458 |
|  | **Nosocomial** | 1.52 | 0.81-2.84 | 0.189 |
|  | **Septic Shock** | 1.01 | 0.42-2.43 | 0.98 |
|  | **MAP (mmHg)** | 0.99 | 0.99-1 | 0.163 |
|  | **Neutropenia** | 1.68 | 0.55-5.1 | 0.36 |
|  | **Platelet count (x 10^9^/L)** | 1 | 0.99-1 | 0.204 |
|  | **Serum Creatinine (mg/dL)** | 0.91 | 0.82-1.02 | 0.116 |
|  | **Serum Bilirubin (mg/dL)** | 1.08 | 0.92-1.27 | 0.327 |
|  | **Serum Albumin (g/dL)** | 1.12 | 0.81-1.56 | 0.485 |
|  | **SOFA** | 1.15 | 1.03-1.27 | 0.011 |
|  | **Transfer to ICU** | 0.22 | 0.12-0.4 | <0.001 |

Complete hierarchical model. aHR: Adjusted Hazard Ratio. CI: Confidence interval. MAP: Mean arterial pressure. SOFA: Sequential Organ Failure Assessment, ICU: Intensive Care Unit
