# Supplementary material for: Survival predictors after intubation in medical wards: A prospective study in 151 patients
Source: PLoS One. 2020 Jun 1;15(6):e0234181. doi: 10.1371/journal.pone.0234181 (PMC7263577; doi:10.1371/journal.pone.0234181)
Supplement: S4 Table — Complete hierarchical model. aHR: Adjusted Hazard Ratio. CI: Confidence interval. MAP: Mean arterial pressure. SOFA: Sequential Organ Failure Assessment, ICU: Intensive Care Unit. (DOCX) [file pone.0234181.s004.docx]

**Supporting Material**

**S4 Table: Multivariate Cox proportional hazards regression analysis for the prediction of 90-day mortality.**

|  | Risk factor | aHR | 95%CI | p |
| --- | --- | --- | --- | --- |
| Block 1 | **Female gender** | 0.93 | 0.65-1.33 | 0.671 |
|  | **Age** | 1.02 | 1-1.03 | **0.007** |
| Block 2 | **Female gender** | 0.87 | 0.61-1.25 | 0.46 |
|  | **Age** | 1.01 | 0.99-1.02 | 0.417 |
|  | **Charlson** | 1.11 | 1.04-1.19 | **0.003** |
| Block 3 | **Female gender** | 0.89 | 0.62-1.29 | 0.546 |
|  | **Age** | 1.01 | 0.99-1.02 | 0.427 |
|  | **Charlson** | 1.08 | 1-1.16 | **0.048** |
|  | **Main Indication** |  |  |  |
|  | **Respiratory** | ref | ref | ref |
|  | **Neurological** | 1.52 | 0.97-2.37 | 0.064 |
|  | **Cardiac arrest** | 1.65 | 0.98-2.78 | 0.059 |
|  | **Location** |  |  |  |
|  | **Emergency Dpt** | ref | ref | ref |
|  | **Ward** | 1.67 | 1-2.77 | **0.05** |
|  | **Other** | 1.75 | 0.72-0.42 | 0.218 |
| Block 4 | **Female gender** | 0.87 | 0.59-1.28 | 0.472 |
|  | **Age** | 1 | 0.99-1.02 | 0.574 |
|  | **Charlson** | 1.08 | 1-1.17 | **0.049** |
|  | **Main Indication** |  |  |  |
|  | **Respiratory** | ref | ref | ref |
|  | **Neurological** | 1.6 | 1.02-2.5 | **0.041** |
|  | **Cardiac arrest** | 1.56 | 0.93-2.63 | 0.094 |
|  | **Location** |  |  |  |
|  | **Emergency Dpt** | ref | ref | ref |
|  | **Ward** | 1.43 | 0.85-2.4 | 0.182 |
|  | **Other** | 1.57 | 0.65-3.84 | 0.318 |
|  | **Circulatory Support** | 2.63 | 1.39-4.96 | **0.003** |
|  | **Infection** |  |  |  |
|  | **No infection** | ref | ref | ref |
|  | **Community** | 1.15 | 0.69-1.91 | 0.592 |
|  | **Nosocomial** | 2.46 | 1.39-4.39 | **0.002** |
|  | **Septic Shock** | 0.45 | 0.19-1.08 | 0.075 |
| Block 5 | **Female gender** | 0.93 | 0.63-1.38 | 0.73 |
|  | **Age** | 1 | 0.98-1.01 | 0.604 |
|  | **Charlson** | 1.2 | 1.1-1.3 | **<0.001** |
|  | **Main Indication** |  |  |  |
|  | **Respiratory** | ref | ref | ref |
|  | **Neurological** | 1.59 | 0.99-2.52 | 0.055 |
|  | **Cardiac arrest** | 1.53 | 0.87-2.7 | 0.143 |
|  | **Location** |  |  |  |
|  | **Emergency Dpt** | ref | ref | ref |
|  | **Ward** | 1.25 | 0.71-2.22 | 0.445 |
|  | **Other** | 1.27 | 0.52-3.12 | 0.597 |
|  | **Circulatory Support** | 2.07 | 1.08-3.96 | **0.029** |
|  | **Infection** |  |  |  |
|  | **No infection** | ref | ref | ref |
|  | **Community** | 0.86 | 0.49-1.51 | 0.606 |
|  | **Nosocomial** | 1.5 | 0.78-2.89 | 0.228 |
|  | **Septic Shock** | 0.68 | 0.28-1.63 | 0.383 |
|  | **MAP (mmHg)** | 0.99 | 0.98-1 | 0.051 |
|  | **Neutropenia** | 1.89 | 0.63-5.67 | 0.259 |
|  | **Platelet count (x 10^9^/L)** | 0.995 | 0.993-0.997 | **<0.001** |
|  | **Serum Creatinine (mg/dL)** | 1.04 | 0.95-1.15 | 0.394 |
|  | **Serum Bilirubin (mg/dL)** | 1.23 | 1.06-1.43 | **0.006** |
|  | **Serum Albumin (g/dL)** | 0.92 | 0.67-1.27 | 0.618 |
| Block 6 | **Female gender** | 0.97 | 0.66-1.42 | 0.862 |
|  | **Age** | 0.99 | 0.98-1.01 | 0.432 |
|  | **Charlson** | 1.16 | 1.07-1.27 | **0.001** |
|  | **Main Indication** |  |  |  |
|  | **Respiratory** | ref | ref | ref |
|  | **Neurological** | 1.24 | 0.76-2.04 | 0.395 |
|  | **Cardiac arrest** | 1.56 | 0.88-2.73 | 0.126 |
|  | **Location** |  |  |  |
|  | **Emergency Dpt** | ref | ref | ref |
|  | **Ward** | 1.14 | 0.64-2.04 | 0.665 |
|  | **Other** | 0.99 | 0.4-2.47 | 0.986 |
|  | **Circulatory Support** | 1.42 | 0.7-2.85 | 0.33 |
|  | **Infection** |  |  |  |
|  | **No infection** | ref | ref | ref |
|  | **Community** | 0.91 | 0.52-1.58 | 0.724 |
|  | **Nosocomial** | 1.64 | 0.86-3.13 | 0.137 |
|  | **Septic Shock** | 0.63 | 0.26-1.48 | 0.285 |
|  | **MAP (mmHg)** | 0.99 | 0.99-1 | 0.14 |
|  | **Neutropenia** | 1.64 | 0.54-4.97 | 0.353 |
|  | **Platelet count (x 10^9^/L)** | 0.997 | 0.995-0.999 | **0.003** |
|  | **Serum Creatinine (mg/dL)** | 0.96 | 0.86-1.08 | 0.491 |
|  | **Serum Bilirubin (mg/dL)** | 1.12 | 0.95-1.32 | 0.177 |
|  | **Serum Albumin (g/dL)** | 0.97 | 0.7-1.34 | 0.848 |
|  | **SOFA** | 1.16 | 1.05-1.28 | **0.004** |
| Block 7 | **Female gender** | 0.9 | 0.61-1.33 | 0.589 |
|  | **Age** | 1 | 0.99-1.02 | 0.936 |
|  | **Charlson** | 1.09 | 0.99-1.19 | 0.074 |
|  | **Main Indication** |  |  |  |
|  | **Respiratory** | ref | ref | ref |
|  | **Neurological** | 0.97 | 0.59-1.6 | 0.918 |
|  | **Cardiac arrest** | 1.2 | 0.68-2.11 | 0.53 |
|  | **Location** |  |  |  |
|  | **Emergency Dpt** | ref | ref | ref |
|  | **Ward** | 1.47 | 0.81-2.64 | 0.206 |
|  | **Other** | 1.75 | 0.68-4.52 | 0.245 |
|  | **Circulatory Support** | 1.09 | 0.55-2.18 | 0.804 |
|  | **Infection** |  |  |  |
|  | **No infection** | ref | ref | ref |
|  | **Community** | 0.78 | 0.44-1.39 | 0.404 |
|  | **Nosocomial** | 1.37 | 0.74-2.56 | 0.321 |
|  | **Septic Shock** | 1.06 | 0.45-2.48 | 0.898 |
|  | **MAP (mmHg)** | 0.99 | 0.98-1 | 0.154 |
|  | **Neutropenia** | 1.78 | 0.6-5.31 | 0.294 |
|  | **Platelet count (x 10^9^/L)** | 0.999 | 0.996-1 | 0.2 |
|  | **Serum Creatinine (mg/dL)** | 0.93 | 0.83-1.04 | 0.184 |
|  | **Serum Bilirubin (mg/dL)** | 1.09 | 0.93-1.28 | 0.274 |
|  | **Serum Albumin (g/dL)** | 0.99 | 0.71-1.37 | 0.945 |
|  | **SOFA** | 1.16 | 1.05-1.29 | **0.003** |
|  | **Transfer to ICU** | 0.25 | 0.15-0.43 | **<0.001** |

Complete hierarchical model. aHR: Adjusted Hazard Ratio. CI: Confidence interval. MAP: Mean arterial pressure. SOFA: Sequential Organ Failure Assessment, ICU: Intensive Care Unit
