# Supplementary material for: Survival predictors after intubation in medical wards: A prospective study in 151 patients
Source: PLoS One. 2020 Jun 1;15(6):e0234181. doi: 10.1371/journal.pone.0234181 (PMC7263577; doi:10.1371/journal.pone.0234181)
Supplement: S5 Table — Complete hierarchical model. aOR: adjusted odds ratio. CI: Confidence interval. GCS: Glasgow Coma Scale, SOFA: Sequential Organ Failure Assessment. (DOCX) [file pone.0234181.s005.docx]

**Supporting Material**

**S5 Table: Multivariate binary logistic regression analysis for the prediction of ICU transfer.**

|  | Risk factor | aOR | 95%CI | p |
| --- | --- | --- | --- | --- |
| Block 1 | **Female gender** | 0.86 | 0.44-1.65 | 0.639 |
|  | **Age** | 0.98 | 0.96-1.004 | 0.122 |
| Block 2 | **Female gender** | 0.8 | 0.41-1.57 | 0.518 |
|  | **Age** | 1 | 0.98-1.03 | 0.904 |
|  | **Charlson score** | 0.81 | 0.7-0.94 | **0.006** |
| Block 3 | **Female gender** | 0.98 | 0.47-2.02 | 0.95 |
|  | **Age** | 1 | 0.97-1.02 | 0.851 |
|  | **Charlson score** | 0.84 | 0.72-0.99 | **0.035** |
|  | **Main Indication** |  |  |  |
|  | **Respiratory** | ref | ref | ref |
|  | **Neurological** | 0.26 | 0.11-0.58 | **0.001** |
|  | **Cardiac arrest** | 0.19 | 0.07-0.51 | **0.001** |
| Block 4 | **Female gender** | 0.89 | 0.42-1.89 | 0.753 |
|  | **Age** | 0.99 | 0.97-1.02 | 0.673 |
|  | **Charlson score** | 0.86 | 0.73-1.02 | 0.079 |
|  | **Main Indication** |  |  |  |
|  | **Respiratory** | ref | ref | ref |
|  | **Neurological** | 0.23 | 0.1-0.53 | **0.001** |
|  | **Cardiac arrest** | 0.15 | 0.05-0.44 | **<0.001** |
|  | **Infection** |  |  |  |
|  | **No infection** | ref | ref | ref |
|  | **Community** | 0.58 | 0.21-1.57 | 0.283 |
|  | **Nosocomial** | 0.32 | 0.12-0.87 | **0.025** |
| Block 5 | **Female gender** | 1.09 | 0.45-2.63 | 0.847 |
|  | **Age** | 1.01 | 0.98-1.05 | 0.416 |
|  | **Charlson score** | 0.76 | 0.62-0.95 | **0.014** |
|  | **Main Indication** |  |  |  |
|  | **Respiratory** | ref | ref | ref |
|  | **Neurological** | 0.26 | 0.08-0.8 | **0.019** |
|  | **Cardiac arrest** | 0.13 | 0.03-0.47 | **0.002** |
|  | **Infection** |  |  |  |
|  | **No infection** | ref | ref | ref |
|  | **Community** | 1.03 | 0.3-3.57 | 0.966 |
|  | **Nosocomial** | 1 | 0.29-3.37 | 0.993 |
|  | **GCS** | 1.14 | 1.02-1.28 | **0.022** |
|  | **Platelet count (x 10^9^/L)** | 1.01 | 1.005-1.014 | **<0.001** |
|  | **Serum Creatinine (mg/dL)** | 0.85 | 0.63-1.16 | 0.304 |
|  | **Serum Bilirubin (mg/dL)** | 0.67 | 0.43-1.07 | 0.092 |
|  | **Serum Albumin (g/dL)** | 1.68 | 0.86-3.27 | 0.131 |
| Block 6 | **Female gender** | 1.05 | 0.43-2.56 | 0.914 |
|  | **Age** | 1.01 | 0.98-1.05 | 0.434 |
|  | **Charlson score** | 0.75 | 0.6-0.94 | **0.012** |
|  | **Main Indication** |  |  |  |
|  | **Respiratory** | ref | ref | ref |
|  | **Neurological** | 0.26 | 0.09-0.82 | **0.021** |
|  | **Cardiac arrest** | 0.13 | 0.03-0.46 | **0.002** |
|  | **Infection** |  |  |  |
|  | **No infection** | ref | ref | ref |
|  | **Community** | 1.04 | 0.3-3.59 | 0.952 |
|  | **Nosocomial** | 0.98 | 0.29-3.35 | 0.978 |
|  | **GCS** | 1.17 | 1.03-1.34 | **0.02** |
|  | **Platelet count (x 10^9^/L)** | 1.01 | 1-1.02 | **<0.001** |
|  | **Serum Creatinine (mg/dL)** | 0.78 | 0.51-1.19 | 0.248 |
|  | **Serum Bilirubin (mg/dL)** | 0.61 | 0.36-1.04 | 0.072 |
|  | **Serum Albumin (g/dL)** | 1.84 | 0.9-3.78 | 0.097 |
|  | **SOFA** | 1.08 | 0.87-1.34 | 0.477 |

Complete hierarchical model. **a**OR: adjusted odds ratio. CI: Confidence interval. GCS: Glasgow Coma Scale, SOFA: Sequential Organ Failure Assessment
