# Supplementary material for: Survival predictors after intubation in medical wards: A prospective study in 151 patients
Source: PLoS One. 2020 Jun 1;15(6):e0234181. doi: 10.1371/journal.pone.0234181 (PMC7263577; doi:10.1371/journal.pone.0234181)
Supplement: S6 Table — Complete hierarchical model. aHR: adjusted hazards ratio. CI: Confidence interval. MAP: mean arterial pressure, SOFA: Sequential Organ Failure Assessment. (DOCX) [file pone.0234181.s006.docx]

**Supporting Material**

**S6 Table: Multivariate cox regression analysis for the mortality of patients transferred to ICU departments.**

|  | Risk Factor | aHR | 95%CI | p |
| --- | --- | --- | --- | --- |
| Block 1 | **Female gender** | 1,05 | 0,57-1,94 | 0,872 |
|  | **Age** | 1,02 | 1-1,04 | **0,04** |
|  | **With spouse** | 1,92 | 0,99-3,75 | 0,056 |
| Block 2 | **Female gender** | 1,11 | 0,59-2,06 | 0,752 |
|  | **Age** | 1,01 | 0,99-1,04 | 0,452 |
|  | **With spouse** | 1,9 | 0,97-3,73 | 0,062 |
|  | **Charlson score** | 1,09 | 0,98-1,23 | 0,125 |
| Block 3 | **Female gender** | 1,24 | 0,65-2,35 | 0,521 |
|  | **Age** | 1 | 0,98-1,03 | 0,943 |
|  | **With spouse** | 1,68 | 0,84-3,38 | 0,144 |
|  | **Charlson score** | 1,1 | 0,97-1,25 | 0,144 |
|  | **Main Indication** |  |  |  |
|  | **Respiratory** | ref | ref | ref |
|  | **Neurological** | 1,02 | 0,5-2,12 | 0,949 |
|  | **Cardiac arrest** | 0,48 | 0,11-2,13 | 0,337 |
|  | **Location** |  |  |  |
|  | **Emergency Dpt** | ref | ref | ref |
|  | **Ward** | 2,28 | 0,87-6 | 0,094 |
|  | **Other** | 5,95 | 1,06-33,42 | **0,043** |
| Block 4 | **Female gender** | 1,52 | 0,72-3,21 | 0,269 |
|  | **Age** | 1,01 | 0,98-1,03 | 0,604 |
|  | **With spouse** | 1,21 | 0,56-2,6 | 0,623 |
|  | **Charlson score** | 1,14 | 0,99-1,31 | 0,075 |
|  | **Main Indication** |  |  |  |
|  | **Respiratory** | ref | ref | ref |
|  | **Neurological** | 1,14 | 0,51-2,54 | 0,752 |
|  | **Cardiac arrest** | 0,45 | 0,1-2,07 | 0,305 |
|  | **Location** |  |  |  |
|  | **Emergency Dpt** | ref | ref | ref |
|  | **Ward** | 1,67 | 0,6-4,59 | 0,324 |
|  | **Other** | 4,26 | 0,67-26,85 | 0,123 |
|  | **Infection** |  |  |  |
|  | **No infection** | ref | ref | ref |
|  | **Community** | 1,27 | 0,52-3,15 | 0,6 |
|  | **Nosocomial** | 5,17 | 1,28-20,89 | **0,021** |
|  | **Septic shock** | 0,168 | 0,02-1,23 | 0,079 |
|  | **Circulatory support** | 9,99 | 2-49,94 | **0,005** |
| Block 5 | **Female gender** | 1,78 | 0,7-4,49 | 0,225 |
|  | **Age** | 0,97 | 0,94-1 | 0,055 |
|  | **With spouse** | 1,94 | 0,78-4,85 | 0,156 |
|  | **Charlson score** | 1,44 | 1,18-1,75 | <0,001 |
|  | **Main Indication** |  |  |  |
|  | **Respiratory** | ref | ref | ref |
|  | **Neurological** | 0,91 | 0,35-2,33 | 0,842 |
|  | **Cardiac arrest** | 0,41 | 0,07-2,32 | 0,316 |
|  | **Location** |  |  |  |
|  | **Emergency Dpt** | ref | ref | ref |
|  | **Ward** | 1,35 | 0,39-4,71 | 0,642 |
|  | **Other** | 1,15 | 0,09-14,65 | 0,946 |
|  | **Infection** |  |  |  |
|  | **No infection** | ref | ref | ref |
|  | **Community** | 0,67 | 0,2-2,16 | 0,498 |
|  | **Nosocomial** | 1,57 | 0,3-8,22 | 0,593 |
|  | **Septic shock** | 0,23 | 0,03-1,7 | 0,148 |
|  | **Circulatory support** | 5,4 | 0,94-30,9 | 0,058 |
|  | **MAP (mmHg)** | 0,98 | 0,96-1 | 0,074 |
|  | **Hematocrit (%)** | 0,97 | 0,92-1,02 | 0,198 |
|  | **Neutropenia** | 3,4 | 0,24-49,9 | 0,365 |
|  | **Platelet count (x 10^9^/L)** | 0,99 | 0,99-1 | **0,002** |
|  | **Serum Creatinine (mg/dL)** | 1,33 | 0,89-2 | 0,17 |
|  | **Serum Bilirubin (mg/dL)** | 1,31 | 0,93-1,84 | 0,13 |
|  | **Serum Albumin (g/dL)** | 0,84 | 0,45-1,57 | 0,575 |
| Block 6 | **Female gender** | 1,79 | 0,67-4,78 | 0,242 |
|  | **Age** | 0,97 | 0,94-1 | 0,109 |
|  | **With spouse** | 1,82 | 0,71-4,69 | 0,212 |
|  | **Charlson score** | 1,34 | 1,09-1,64 | **0,05** |
|  | **Main Indication** |  |  |  |
|  | **Respiratory** | ref | ref | ref |
|  | **Neurological** | 0,61 | 0,23-1,67 | 0,34 |
|  | **Cardiac arrest** | 0,38 | 0,07-1,99 | 0,252 |
|  | **Location** |  |  |  |
|  | **Emergency Dpt** | ref | ref | ref |
|  | **Ward** | 1,2 | 0,35-4,15 | 0,778 |
|  | **Other** | 0,88 | 0,08-9,41 | 0,916 |
|  | **Infection** |  |  |  |
|  | **No infection** | ref | ref | ref |
|  | **Community** | 1,01 | 0,29-3,52 | 0,986 |
|  | **Nosocomial** | 2,02 | 0,39-10,45 | 0,403 |
|  | **Septic shock** | 0,12 | 0,02-0,9 | **0,04** |
|  | **Circulatory support** | 4,7 | 0,86-25,9 | 0,075 |
|  | **MAP (mmHg)** | 0,98 | 0,97-1 | 0,084 |
|  | **Hematocrit (%)** | 0,97 | 0,92-1,02 | 0,258 |
|  | **Neutropenia** | 4,01 | 0,29-57,9 | 0,298 |
|  | **Platelet count (x 10^9^/L)** | 0,99 | 0,99-1 | 0,126 |
|  | **Serum Creatinine (mg/dL)** | 1,07 | 0,68-1,69 | 0,766 |
|  | **Serum Bilirubin (mg/dL)** | 1,16 | 0,8-1,68 | 0,442 |
|  | **Serum Albumin (g/dL)** | 1,03 | 0,54-1,96 | 0,934 |
|  | **SOFA** | 1,24 | 1,02-1,51 | **0,03** |
| Block 7 | **Female gender** | 1,81 | 0,67-4,89 | 0,242 |
|  | **Age** | 0,97 | 0,94-1,01 | 0,109 |
|  | **With spouse** | 1,82 | 0,7-4,69 | 0,213 |
|  | **Charlson score** | 1,34 | 1,09-1,65 | **0,006** |
|  | **Main Indication** |  |  |  |
|  | **Respiratory** | ref | ref | ref |
|  | **Neurological** | 0,62 | 0,23-1,68 | 0,344 |
|  | **Cardiac arrest** | 0,37 | 0,06-2,12 | 0,264 |
|  | **Location** |  |  |  |
|  | **Emergency Dpt** | ref | ref | ref |
|  | **Ward** | 1,19 | 0,34-4,14 | 0,783 |
|  | **Other** | 0,92 | 0,08-10,77 | 0,944 |
|  | **Infection** |  |  |  |
|  | **No infection** | ref | ref | ref |
|  | **Community** | 1,02 | 0,29-3,56 | 0,982 |
|  | **Nosocomial** | 1,97 | 0,32-10,7 | 0,432 |
|  | **Septic shock** | 0,123 | 0,01-1,29 | 0,081 |
|  | **Circulatory support** | 4,5 | 0,69-29,6 | 0,117 |
|  | **MAP (mmHg)** | 0,98 | 0,97-1 | 0,088 |
|  | **Hematocrit (%)** | 0,97 | 0,92-1,03 | 0,296 |
|  | **Neutropenia** | 4,43 | 0,22-89,4 | 0,332 |
|  | **Platelet count (x 10^9^/L)** | 0,997 | 0,994-1,001 | 0,139 |
|  | **Serum Creatinine (mg/dL)** | 1,09 | 0,633-1,88 | 0,757 |
|  | **Serum Bilirubin (mg/dL)** | 1,17 | 0,79-1,73 | 0,446 |
|  | **Serum Albumin (g/dL)** | 1,04 | 0,53-2,05 | 0,911 |
|  | **SOFA** | 1,24 | 0,99-1,55 | 0,064 |
|  | **Transfer >1 day after intubation** | 0,92 | 0,2-4,1 | 0,911 |

Complete hierarchical model. aHR: adjusted hazards ratio. CI: Confidence interval. MAP: mean arterial pressure, SOFA: Sequential Organ Failure Assessment
